# Receptor-like kinase BAM1 facilitates early movement of the Tobacco mosaic virus

**DOI:** 10.1038/s42003-021-02041-0

**Published:** 2021-04-30

**Authors:** Phu-Tri Tran, Vitaly Citovsky

**Affiliations:** Department of Biochemistry and Cell Biology, State University of New York, Stony Brook, NY USA

**Keywords:** Virus-host interactions, Viral pathogenesis

## Abstract

Cell-to-cell movement is an important step for initiation and spreading of virus infection in plants. This process occurs through the intercellular connections, termed plasmodesmata (PD), and is usually mediated by one or more virus-encoded movement proteins (MP) which interact with multiple cellular factors, among them protein kinases that usually have negative effects on MP function and virus movement. In this study, we report physical and functional interaction between MP of *Tobacco mosaic virus* (TMV), the paradigm of PD-moving proteins, and a receptor-like kinase BAM1 from Arabidopsis and its homolog from *Nicotiana benthamiana*. The interacting proteins accumulated in the PD regions, colocalizing with a PD marker. Reversed genetics experiments, using BAM1 gain-of-function and loss-of-function plants, indicated that BAM1 is required for efficient spread and accumulation the virus during initial stages of infection of both plant species by TMV. Furthermore, BAM1 was also required for the efficient cell-to-cell movement of TMV MP, suggesting that BAM1 interacts with TMV MP to support early movement of the virus. Interestingly, this role of BAM1 in viral movement did not require its protein kinase activity. Thus, we propose that association of BAM1 with TMV MP at PD facilitates the MP transport through PD, which, in turn, enhances the spread of the viral infection.

## Abstract (web summary)

Tran and Citovsky show that receptor-like kinase BAM1 is required for efficient spread and the accumulation of the virus during initial stages of infection of *Arabidopsis* and *Nicotiana* by Tobacco mosaic virus (TMV). This study provides insights into the TMV’s transmission mechanism in plants.

## Introduction

Plant viruses have evolved various mechanisms to spread their genomes from an initially infected cell to neighboring cells, thereby allowing local and systemic spread of viruses in plants. The local spreading of viruses known as cell-to-cell movement is generally mediated by one or more movement proteins (MPs), which exploit cell-to-cell connectivity of plasmodesmata (PD) and transport the viral genomic nucleic acid into the adjacent cells^1^. A large proportion of viral MPs have been classified into the 30 K superfamily due to their homologies with the 30 kDa MP of the *Tobacco mosaic virus* (TMV), the first virus discovered^2–4^. While some of the 30 K MPs, commonly found among members of the families *Comoviridae*, *Bunyaviridae*, *Caulimoviridae*, and *Bromoviridae*, act by producing tubules that protrude through PD and mediate virus movement as intact virions^5^, many others are not associated with tubule formation and traffic viral RNA as ribonucleoprotein complexes^6^. In a model for non-tubule-mediated movement of viral ribonucleoprotein complexes, TMV MP binds single-stranded nucleic acids^7^, localizes to PD and increases their permeability^8,9^, and interacts with numerous cellular factors, including protein kinases that phosphorylate MP^10^.

BAM1 is a receptor-like kinase (RLK) required for meristem function^11^ and early anther development in Arabidopsis^12^. Also, BAM1 is known to facilitate movement of silencing signals in Arabidopsis and to be targeted by C4^13^, a small protein of *Tomato yellow leaf curl virus* involved in gene silencing and viral movement^14^. Whether the BAM1 is recognized by the 30 K viral MPs and participates in their function has not been examined. Here we report direct interaction between BAM1 from two plant species, *Arabidopsis thaliana* and *Nicotiana benthamiana*, and TMV MP and describe the involvement of BAM1 in the early stages of TMV spread and TMV MP cell-to-cell movement.

## Results

### TMV MP interacts with BAM1

**Fig. 1:**
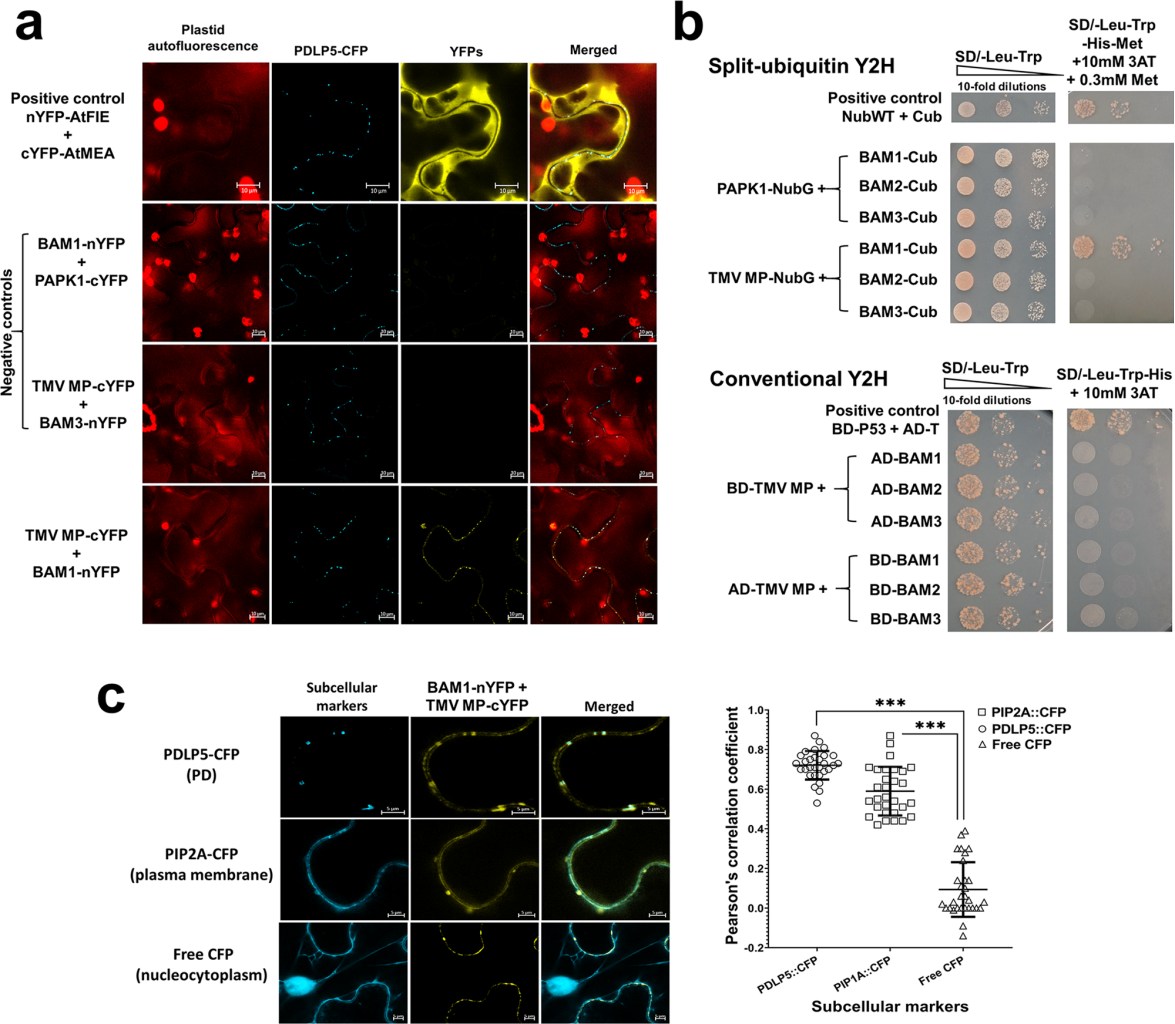
BAM1 interacts with TMV MP. **a** Interaction in living plant cells. Protein interaction was analyzed by BiFC in *N. benthamiana* leaves agroinfiltrated with the indicated combinations (1:1 w/w ratio) of the tested expression constructs and an expression construct for PDLP5-CFP as a PD marker. YFP signal is in yellow; CFP signal is in cyan. Images were recorded 48 h after agroinfiltration and are single confocal sections; all images are representative of multiple independent experiments (*N* = 20 images from 5 plants). Scale bars = 10 µm. **b** Interaction in yeast. Protein interaction was analyzed by Y2H split-ubiquitin and conventional Y2H approaches. Cell growth in the absence of leucine and tryptophan represents non-selective conditions for the indicated combinations of the tested expression constructs. Cell growth in the absence of leucine, tryptophan, and histidine represents selective conditions for protein–protein interaction. **c** Quantitative analyses of colocalization correlation between the BAM1-MP BiFC complexes and the PD marker PDLP5-CFP, plasma membrane marker PIP2A-CFP, or the nucleocytoplasmic marker free CFP. Scale bars = 5 µm. Pearson’s correlation coefficients (PCC) of YFP and CFP signals were generated from 30 regions of interest using the ImageJ software (https://imagej.nih.gov/ij/). Error bars represent standard deviation of these 30 regions of interest. Asterisks indicate statistically significant differences between colocalization of BAM1-MP BiFC complexes with the PDLP5, PIP2A, or CFP markers, ***P < 0.001.

To examine whether TMV MP interacts with BAM1 in planta, we utilized the bimolecular fluorescence complementation (BiFC) assay, in which TMV MP and BAM1 were fused to the C-and N-terminal halves of yellow fluorescent protein (YFP), i.e., nYFP and cYFP, respectively, and coexpressed in *N. benthamiana* leaf epidermis together with the PD marker PDLP5^15–18^ tagged with cyan fluorescent protein (CFP). These experiments showed that TMV MP interacted with BAM1 at the PD areas, colocalizing with PDLP5 (Fig. 1a, c). This interaction was specific because, in negative control experiments, no BiFC was observed between TMV MP and the Arabidopsis plasma membrane-localizing RLK BAM3^19^ or between BAM1 and the Arabidopsis PD-localizing protein PAPK1 (Fig. 1a). In positive control experiments and supporting previous observations^20^, Arabidopsis proteins FIE and MEA interacted with each other (Fig. 1a).

Further quantitative analyses of localization, using PDLP5-CFP as the PD marker and free CFP as the nucleocytoplasmic marker, confirmed that the interacting MP–BAM1 complexes localized at or near PD and plasma membrane but not in the cell nucleus or cytoplasm (Fig. 1c). Specifically, MP-cYFP/BAM1-nYFP complexes displayed the typical PD-specific punctate accumulation at the cell membrane, co-occurring with PDLP5-CFP at PD (Pearson correlation coefficient [PCC] of YFP/CFP signal overlap 0.72 ± 0.07) and with PIP2A at plasma membrane (PPC 0.59 ± 0.12), whereas no significant signal overlap was observed with free CFP (PCC 0.09 ± 0.13) (Fig. 1c and Table S2).

Next, we confirmed the TMV MP–BAM1 interaction in an independent assay, using a yeast two-hybrid (Y2H) system. Because our BiFC data indicated interaction at PD, we utilized a membrane-based split-ubiquitin system, in which TMV MP was fused to the N-terminal half of the ubiquitin molecule (NubWT) with an isoleucine-13-to-glycine mutation (NubG) that prevents spontaneous reassembly of the ubiquitin halves^21^ and BAM1 was tagged with the C-terminal half of ubiquitin fused with a reporter transcription factor (Cub); the N-terminal half of the wild-type ubiquitin was used as positive control^21^. Figure 1b shows that, as expected, NubWT and Cub reconstructed the ubiquitin molecule, resulting in its cleavage, release of the reporter, and induction histidine prototrophy and cell growth. In negative controls, coexpression of either BAM1-Cub and PAPK1-NubG or TMV MP-NubG and BAM3-Cub failed to allow cell growth, indicating the lack of interaction. However, TMV MP-NubG and BAM1-Cub interacted with each other and promoted cell growth in the absence of histidine (Fig. 1b, upper panel). Interestingly, the TMV MP–BAM1 interaction did not occur in an ectopic intracellular location, i.e., the nucleus, when these proteins were expressed in a conventional Y2H system where the interacting molecules are imported into the cell nucleus (Fig. 1b, lower panel). Thus, the membrane/PD location may be more conducive to the MP–BAM1 interaction. In control experiments, none of the tested protein combinations interfered with cell growth under non-selective conditions (i.e., in the presence of histidine) (Fig. 1b). The specificity of the recognition of TMV MP by BAM1 was further demonstrated by the observations that BAM1 did not interact with any of the other TMV-encoded proteins. Specifically, in addition to MP, TMV encodes two other main types of proteins, a coat protein (CP) and an RNA-dependent RNA polymerase (RdRp)^22,23^. Figure S3 shows that BAM1 was unable to interact with any of the four RdRP domains^23^ or with CP both in the BiFC and split-ubiquitin Y2H systems. The specificity of recognition of BAM1 by TMV MP was narrow because TMV MP, surprisingly, did not interact in the split-ubiquitin Y2H system with BAM2 (Fig. 1b), the closest homolog of BAM1 in the Arabidopsis genome^13^.

**Fig. 2:**
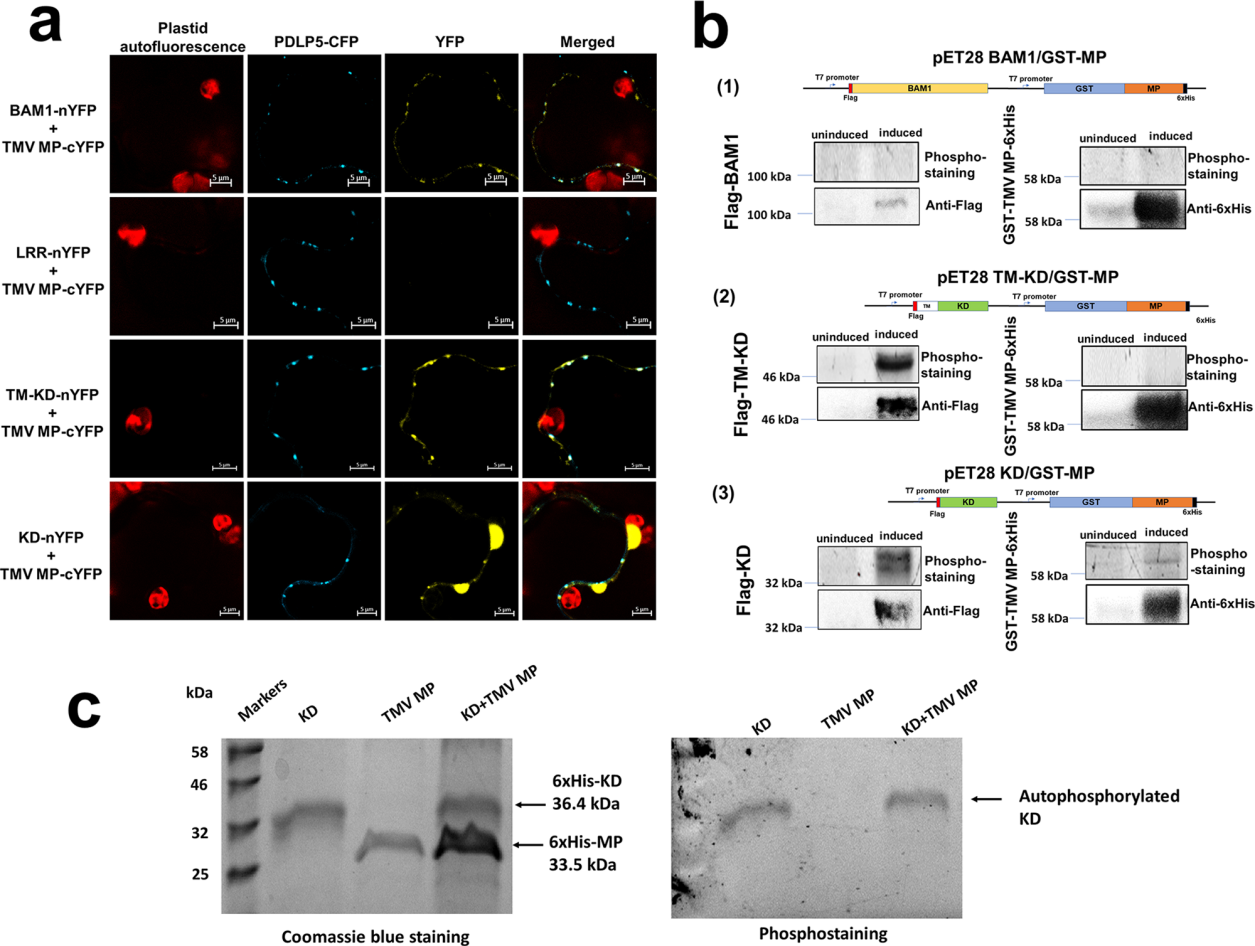
Interaction of the TM-KD and KD domains of BAM1 with TMV MP and phosphorylation of TMV MP. **a** Interaction. Protein interaction was analyzed by BiFC in *N. benthamiana* leaves agroinfiltrated with the indicated combinations (1:1 w/w ratio) of the tested expression constructs and an expression construct for PDLP5-CFP as a PD marker. YFP signal is in yellow; CFP signal is in cyan. Images were recorded 48 h after agroinfiltration and are single confocal sections; images are representative of multiple independent experiments (*N* = 20 images from 5 plants). Scale bars = 5 µm. **b** Transphosphorylation. Uninduced *E. coli* cells or cells induced with IPTG to coexpress GST-MP-6His and Flag-BAM1 (1), Flag-TM-KD (2), or Flag-KD (3) were analyzed using the transphosphorylation assay followed by phosphostaining, and protein expression was monitored by western blotting with anti-6xHis or anti-Flag antibodies, respectively. Schematic illustrations of the specific dual expression constructs used in each experiment are shown. **c** In vitro phosphorylation. Indicated combinations of recombinant KD and TMV MP were analyzed using the in vitro phosphorylation assay followed by phosphostaining (right panel) and Coomassie blue staining (left panel). Protein molecular mass markers are shown in kilodaltons.

We then examined the BAM1–TMV MP interaction in more detail. BAM1 has three functional/structural domains: leucine-rich repeats (LRRs), transmembrane domain (TM), and protein kinase catalytic domain (KD) (Fig. S1a). Thus, we assayed each of them for the ability to bind TMV MP in plant cells, using BiFC. The LRR domain tagged with nYFP was unable to bind TMV MP-cYFP, whereas, in positive control, the full-length BAM1-nYFP interacted with TMV MP-cYFP at PD, colocalizing with the PD marker PDLP5-CFP (Fig. 2a). The ability of BAM1 to interact with TMV MP at PD most likely was contained within its C-proximal part that comprises both the TM and KD domains (Fig. S1a). Figure 2a shows the BAM1 mutant, composed of the TM-KD domains and tagged with nYFP, interacted with TMV MP-cYFP, and that the interacting complexes colocalized with PDLP5-CFP. The catalytic domain alone, KD-nYFP, still interacted with TMV MP-cYFP, yet this interaction largely lost its PD-specific localization (Fig. 2a).

**Fig. 3:**
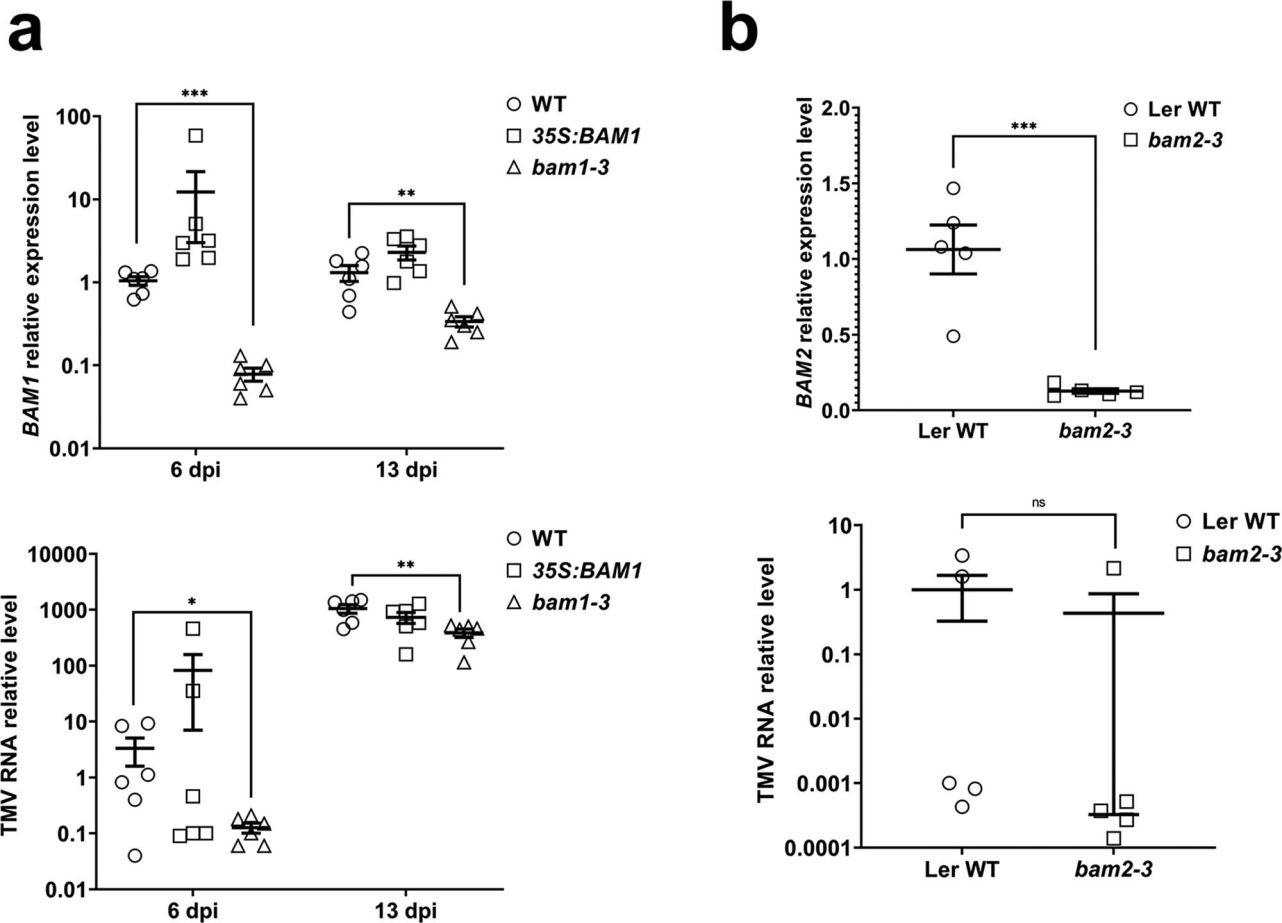
TMV spread in *BAM1* gain-of-function and loss-of-function mutants. **a** RT-qPCR analysis of BAM1 mutants. Relative expression levels of *BAM1* (upper panel) and relative levels of TMV genomic RNA (lower panel) in systemic leaves of the wild type, gain-of-function (*35S:BAM1*), and loss-of-function *Arabidopsis* Col-0 lines (*bam1-3*) at 6 and 13 dpi. **b** RT-qPCR analysis of BAM2 mutants. Upper panel: relative expression levels of *BAM2* in the leaves of the loss-of-function *Arabidopsis* Ler-0 line (*bam2-3*). Lower panel: relative levels of TMV genomic RNA in the systemic leaves of the loss-of-function *Arabidopsis* Ler-0 line (*bam2-3*) at 6 dpi. Error bars in **a**, **b** represent standard error of six and five biological replicates, respectively. Asterisks indicate statistically significant differences between the tested plants and the control wild-type (WT) plants; *P < 0.05, **P < 0.01, and ***P < 0.001; ns not statistically significant (*P* ≥ 0.05).

Finally, we examined whether BAM1 and/or its separate domains could phosphorylate TMV MP. To this end, we utilized a transphosphorylation assay in *Escherichia coli* cells, previously used to characterize diverse plant receptor kinases, such as BAK1, PEPR1, and FLS2^24^, as well as human protein kinase A^25^. We coexpressed inducibly from the same plasmid construct glutathione *S*-transferase (GST)-tagged TMV MP together with BAM1 or its LRR, TM-KD, and KD domains; in addition, the coexpressed proteins were tagged by different epitopes to allow detection of their expression (Figs. 2b and S2). Following induction, the proteins were allowed to coexpress and the phosphorylation reaction to occur for 16 h, followed by denaturing gel electrophoresis and phosphoprotein gel staining. This analysis revealed that TMV MP, TM-KD, and KD were expressed well, whereas BAM1 showed only very weak expression. Potentially due to this low expression, we were unable to detect the protein kinase activity of BAM1 (Fig. 2b-1). In contrast, both TM-KD and KD exhibited a clear ability to autophosphorylate (Fig. 2b-2, 3), yet only KD phosphorylated TMV MP, albeit weakly (Fig. 2b-3), and TM-KD did not (Fig. 2b-2). TMV MP phosphorylation by KD alone most likely did not reflect the biological interaction because the KD–TMV MP complexes largely mislocalized in the cell (Fig. 2a). That TMV MP does not represent the efficient substrate of KD is suggested further by observations that, when both proteins were expressed and isolated from *E. coli*, the recombinant KD failed to phosphorylate the recombinant TMV MP in vitro (Fig. 2c). Based on these data, we hypothesize that the BAM1–TMV MP interaction and its potential role in TMV MP function and/or PD transport most likely does not involve phosphorylating the MP molecule.

### BAM1 is required for optimal spread of TMV in Arabidopsis

To investigate the involvement of BAM1 in TMV spread in Arabidopsis, we utilized the previously described *35S:BAM1* gain-of-function Arabidopsis plants^13^ and the *bam1-3* loss-of-function Arabidopsis allele^26^. Quantitative reverse transcription polymerase chain reaction (RT-qPCR) analysis of these plant lines showed that the *35S:BAM1* and *bam1-3* plants at 6 days post inoculation (dpi) accumulated ca. 12-fold higher and ca. 10-fold lower amounts of *BAM1* transcripts, respectively, than the wild-type plants; at 13 dpi, those accumulations were, respectively, ca. twofold higher and ca. threefold lower than the wild-type (Fig. 3a and Table S3a). These plants were inoculated mechanically with TMV, and accumulation of the viral RNA in the upper, uninoculated leaves was monitored by RT-PCR at 6 and 13 dpi. Figure 3a shows that the relative levels of TMV RNA at 6 dpi were ca. 21 times higher in the *35S:BAM1* gain-of-function plants and ca. 25 times lower in the *bam1-3* loss-of-function mutant. At 13 dpi, however, the differences in TMV RNA levels between the wild type and *35S:BAM1* lines became statistically insignificant, whereas the levels of TMV RNA in the *bam1-3* mutant, although remaining depressed in a statistically significant fashion, were only ca. threefold lower than those of the wild-type plants (Fig. 3a and Table S3b). These observations suggest that BAM1 plays a role in the systemic infection of TMV and that the main effect of BAM1 is exerted at an early stage of the viral spread. Confirming the specific involvement of BAM1 in viral spread, and consistent with the inability of TMV MP to interact with BAM2 (Fig. 1), we observed no statistically significant effects of the loss of function of BAM2 on systemic TMV infection at 6 and 13 dpi in the *bam2-3* Arabidopsis plants^11^ (Fig. 3b and Table S4).

### BAM1 is involved in cell-to-cell movement of TMV MP in *N. benthamiana*

**Fig. 4:**
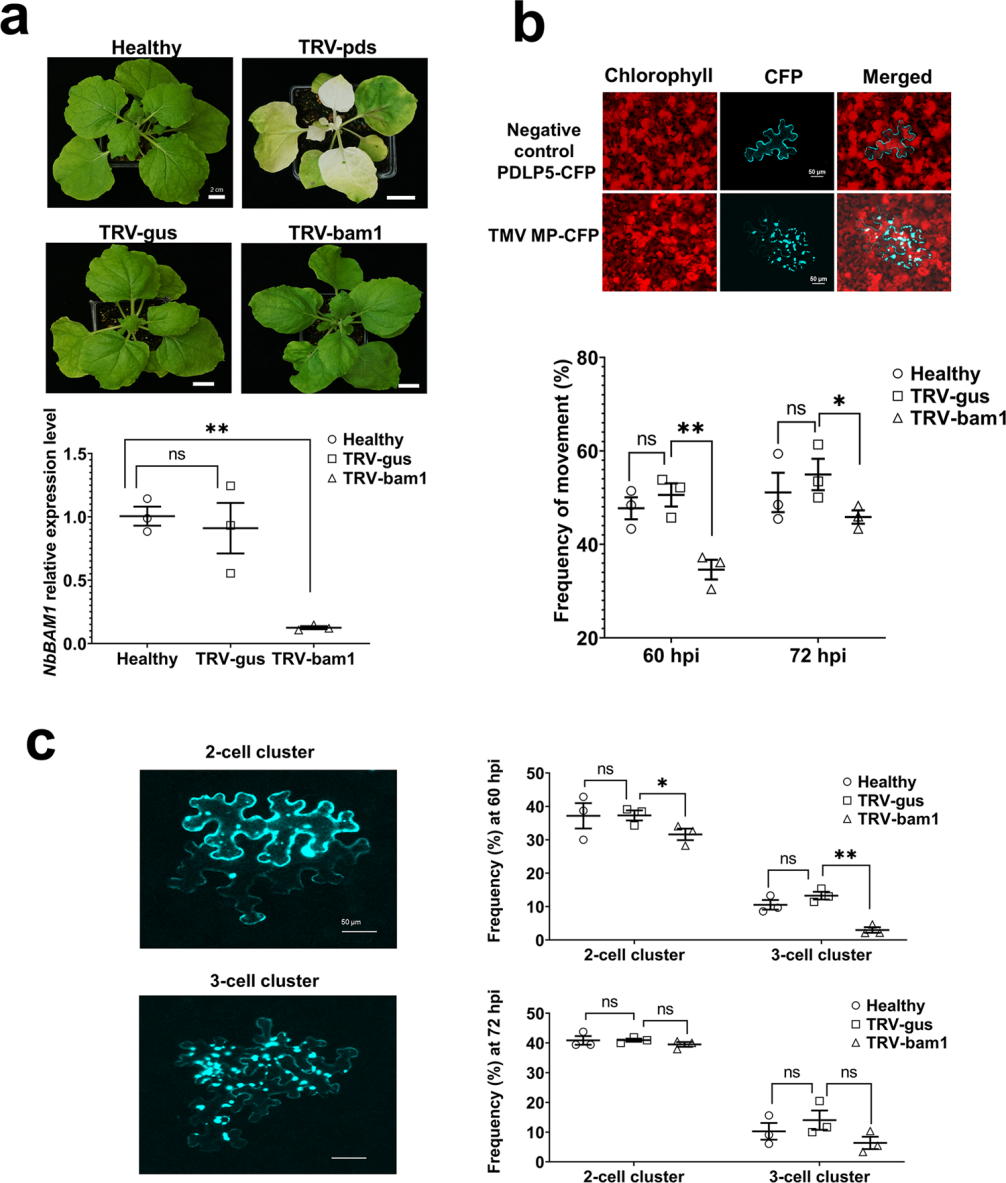
Effect of VIGS of *NbBAM1* on cell-to-cell movement of TMV MP. **a** Chlorotic phenotype and expression levels of *NbBAM1* in *PDS-* and *NbBAM1*-silenced *N. benthamiana* plants 2 weeks after treatment with TRV vectors*. BAM1* expression was quantified by RT-qPCR. Healthy, intact plants; TRV-pds, plants infected with *PDS*-specific TRV VIGS construct; TRV-bam1, plants infected with *BAM1*-specific TRV VIGS construct; TRV-gus, plants infected with control *GUS*-specific TRV VIGS construct. **b** Cell-to-cell movement of TMV MP in *NbBAM1*-silenced *N. benthamiana* plants. Constructs expressing MP-CFP, or PDLP5-CFP as a cell-autonomous control, were agroinfiltrated into *N. benthamiana* leaf tissues. Images were recorded 60 h after agroinfiltration and are single confocal sections representative of multiple independent experiments (*N* = 20 images from 5 plants). Scale bars = 50 µm. Frequency of MP-CFP cell-to-cell movement was quantified by scoring CFP signal-containing cell clusters at 60 h post inoculation (hpi) and 72 hpi of the MP-CFP expression constructs onto the control (TRV-gus) and *NbBAM1* silenced plants (TRV-bam1). **c** Extent of cell-to-cell movement of TMV MP in *NbBAM1*-silenced *N. benthamiana* plants. Construct expressing MP-CFP was agroinfiltrated into *N. benthamiana* leaf tissue. Images were recorded at 60 hpi and are single confocal sections representative of multiple independent experiments (*N* = 20 images from 5 plants). Scale bars = 50 µm. The extent of MP-CFP cell-to-cell movement was scored as frequency of cell clusters containing the CFP signal and composed of 2 or 3 cells/cluster. TRV-gus, control plants; TRV-bam1, *NbBAM1*-silenced plants. Error bars represent standard error of three biological replications. Asterisks indicate statistically significant differences between the silenced plants and the control wild-type (WT) plants; *P < 0.05 and **P < 0.01.

The effect of BAM1 on TMV movement may derive from its involvement in the function of TMV MP. We tested this notion by comparing the extent of cell-to-cell movement of CFP-tagged MP in wild-type and BAM1-deficient plants. For these experiments, we elected to use *N. benthamiana*, the host of choice for many plant virus movement studies whose relatively large leaves allow more precise detection of cell-to-cell movement, and generated *BAM1* knock-down plants using virus-induced gene silencing (VIGS). To this end, a full-length cDNA of *N. benthamiana* homolog of *BAM1* (*NbBAM1*) was isolated (Fig. S1a, see GenBank accession number MT623393); its amino acid sequence analysis indicated 85% homology and 77% identity with the Arabidopsis BAM1 (Fig. S1b), which phylogenetically belongs to a related clade (Fig. S1c). Unlike Arabidopsis, the Solanaceae family, which includes *N. benthamiana*, does not encode BAM2; thus, we focused on NbBAM1. The ability of NbBAM1 to interact with TMV MP was confirmed by BiFC, in which the interacting proteins colocalized with the PDLP5 PD marker (Fig. S4a). For VIGS, a short fragment of *NbBAM1* was cloned into a tobacco rattle virus (TRV)-based vector, and the resulting VIGS construct (TRV-bam1) was inoculated on *N. benthamiana* plants. Two weeks after inoculation, no statistically significant differences were found in *BAM1* expression between the non-treated (healthy) plants and plants inoculated with a negative control VIGS construct (TRV-gus) (Fig. 4a). Thus, the TRV sequences in the VIGS constructs did not affect the native expression levels of *BAM1*. In a positive control, silencing of the *N. benthamiana* phytoene desaturase (*PDS*) gene by the TRV-pds VIGS construct resulted in a typical bleaching phenotype characteristic for leaves with reduced amounts of carotenoids associated with suppression of PDS expression^27,28^ (Fig. 4a). Similarly, the *BAM1*-specific VIGS construct (TRV-bam1) silenced ca. 90% of *BAM1* expression (Fig. 4a and Table S5a).

Next, we used the *BAM1*-silenced plants (TRV-bam1 plants) to assess whether they support the cell-to-cell movement of TMV MP. Figure 4b shows that, in negative control experiments, expression of a CFP-tagged Arabidopsis PDLP5 protein known to associate with PD, but not to move between cells^15–18^, produced only single cells with the CFP signal. In contrast, TMV MP-CFP exhibited cell-to-cell movement. TMV MP-CFP transport was scored as appearance of two-cell or three-cell clusters containing the CFP signal, with the initially expressing cells exhibiting higher signal intensity than their neighboring cells (Fig. 4b, c). Specifically, in the control TRV-gus plants, we observed TMV MP-CFP-containing clusters in ca. 50% of the expressing cells. The extent of the TMV MP-CFP cell-to-cell movement in the TRV-bam1 plants was significantly lower, i.e., ca. 35%; this reduced cell-to-cell movement of TMV MP-CFP remained detectable at 72 dpi, i.e., ca. 55 vs 45% (Fig. 4b and Table S5b). Interestingly, the main inhibitory effect of *BAM1* silencing was observed on the more extensive TMV MP-CFP movement, i.e., the formation of 3-cell clusters, which was reduced from 13 to 2% in the TRV-gus and TRV-bam1 plants, respectively (Fig. 4c and Table S5c). Collectively, these data suggest that BAM1 is required for optimal movement of TMV MP between cells.

### BAM1 is required for optimal spread of TMV in *N. benthamiana*

**Fig. 5:**
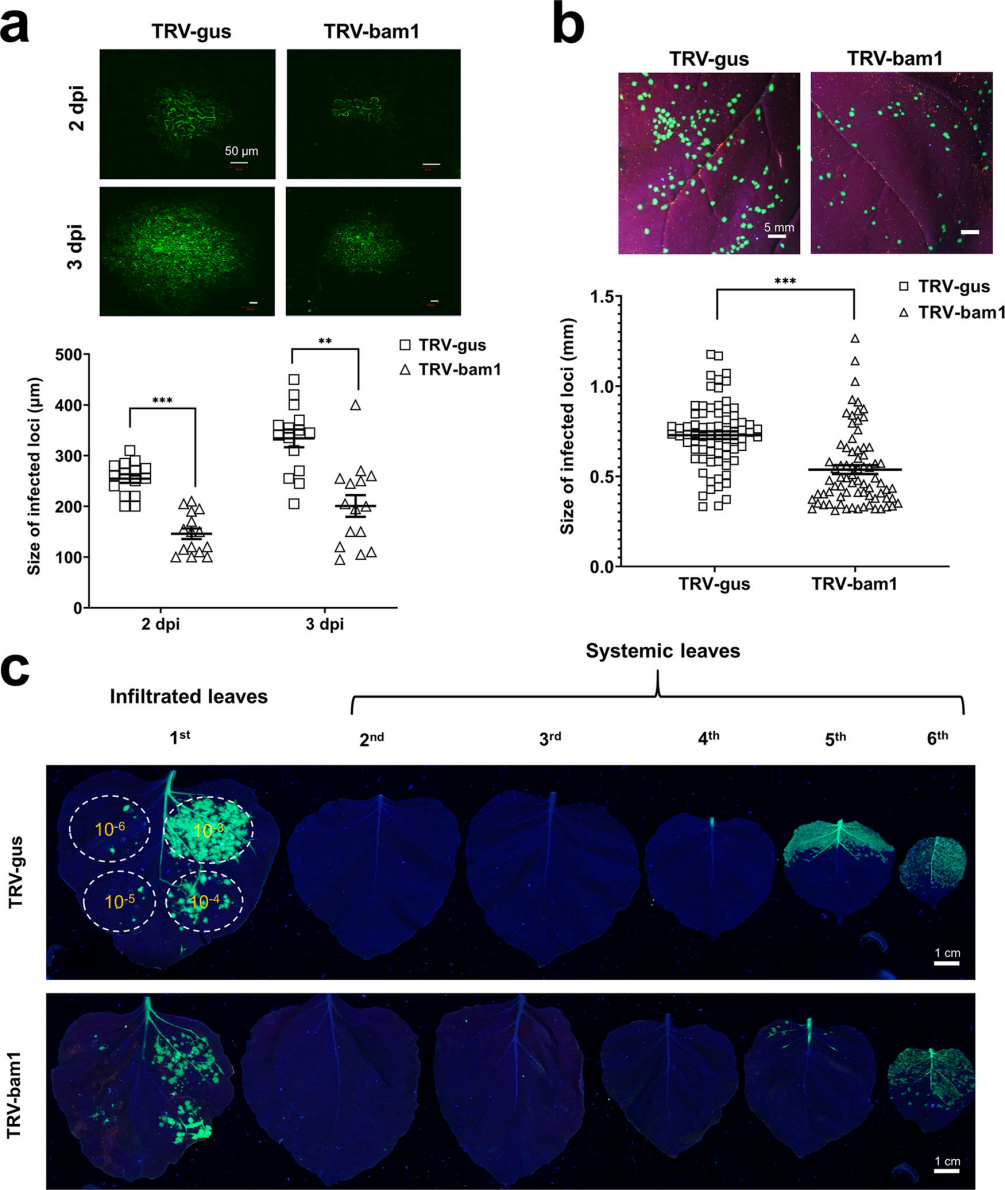
Effect of VIGS of *NbBAM1* on cell-to-cell and systemic movement of TMV. **a** Effect on cell-to-cell movement of TMV lacking CP. Control (TRV-gus) and *NbBAM1*-silenced plants (TRV-bam1) were inoculated with the pTRBO-G construct, and the GFP signal in the inoculated leaves was recorded at 2 and 3 dpi; images are representative of multiple independent experiments (*N* = 20 images from 5 plants). Scale bars = 50 µm. Viral movement within the inoculated leaves was quantified by measuring average diameters of GFP-expressing infection loci at each time point using the ImageJ software. **b** Effect on cell-to-cell movement of TMV. Control (TRV-gus) and *NbBAM1*-silenced plants (TRV-bam1) were inoculated with the pTRBO-CP-G construct, and the GFP signal in the inoculated leaves was recorded at 3 dpi; images are representative of multiple independent experiments (*N* = 20 images from 5 plants). Scale bars = 5 mm. Viral movement within the inoculated leaves was quantified by measuring the size of GFP-expressing infection loci using ImageJ. Error bars in **a**, **b** represent standard error of 15 and 75 biological replicates, respectively; asterisks indicate statistically significant differences between the TRV-bam1 plants and the control TRV-gus plants; **P < 0.01 and ***P < 0.001. **c** Effect on systemic movement of TMV. Control (TRV-gus) and *NbBAM1*-silenced plants (TRV-bam1) were inoculated with the pTRBO-CP-G construct; the inoculum was applied at a dynamic range of the indicated serial dilutions (OD_600_ from 10^−3^ to 10^−6^). The GFP signal in the inoculated (first leaf) and uninoculated, systemic leaves (second to sixth leaf) was recorded at 10 dpi; images are representative of multiple independent experiments (*N* = 20 images from 5 plants). Scale bars = 1 cm.

We examined the effects of *BAM1* silencing on local and systemic movement of TMV. To focus on the local movement, we utilized a TMV mutant^29^ that contains functional MP but lacks the viral CP known to be required for viral systemic spread^30^. An infectious TMV construct without CP and expressing a green fluorescent protein (GFP) reporter (TRBO-G) was inoculated onto the *BAM1*-silenced plants, and the viral movement was monitored by confocal microscopy. Figure 5a shows that, in the control, TRV-gus plants, the TMV local spread within the inoculated leaf was easily detectible already at 2 dpi, and it increased substantially at 3 dpi. The extent of this TMV movement was substantially decreased in the TRV-bam1 plants. Quantification of the size of the GFP signal foci revealed, with statistical significance, that silencing of the *BAM1* reduced cell-to-cell spread areas of TMV from 240 to 150 μm at 2 dpi and from 350 to 200 μm at 3 dpi or by 38 and 43%, respectively (Fig. 5a and Table S6a).

Next, we investigated whether the *BAM1* silencing affects the cell-to-cell movement of encapsidated TMV virions. We generated a modified infectious TRBO-G construct that also encodes the viral CP. Virions derived from this construct, designated TRBO-CP-G, were mechanically inoculated onto the TRV-gus and TRV-bam1 plants. At 3 dpi, the GFP-expressing infection foci of variable sizes were clearly visible on the inoculated leaves (Fig. 5b). Size distribution of the increasing sizes of these foci, indicated that the local viral movement in *BAM1*-silenced plants was impaired compared to the control, non-silenced plants (Fig. 5b and Table S6b).

We then analyzed in more detail the effects of *BAM1* silencing on local- and long-distance movement of TMV virions. TMV local movement occurs within the inoculated issue, whereas, for its systemic movement, the virus invades the host vasculature and unloads within the systemic organs, paralleling transport of photoassimilates from the source to sink tissues, and then spreads locally in the systemic tissues^31^. The TRBO-CP-G construct was inoculated onto the TRV-gus and TRV-bam1 plants. At 10 dpi, the lower, inoculated and the upper, uninoculated leaves were detached and examined for the presence and distribution of the GFP signal. Figure 5c shows that, in the control TRV-gus plants, the virus spread efficiently in the inoculated areas, with GFP signal paralleling the size of the viral inoculum. In the uninoculated leaves, the virus traveled to the sink apical leaves, displaying efficient infection of and local movement within leaves 5 and 6; leaves 2–4 showed virtually no visible signs of the virus presence. In the TRV-bam1 plants, the local viral movement was compromised, with higher inocula of the virus clearly exhibiting limited spread of the GFP signal. The systemic movement of the virus in these plants was not detectibly affected, i.e., it was detected in the same sink leaves as in the control plants; the local viral movement in these leaves was compromised, especially in leaf 5 (Fig. 5c). Thus, *BAM1* silencing interferes with the local spread of the virus.

Finally, we used the *BAM1*-silenced plants to confirm the apparent lack of BAM1 ability to phosphorylate TMV MP (Fig. S4b). To this end, we utilized the observations that purified TMV MP is phosphorylated in a cell-free system by cell wall-enriched fractions of plant cells^32^. We further calibrated this assay to identify the optimal amount of ATP required for the reaction (Figs. S4b-1) and used it to assay TMV MP phosphorylation by cell wall-enriched fractions of the TRV-bam1 and TRV-gus plants. Figure S4b-2 shows that TRV-bam1 cell walls phosphorylated TMV MP to the same overall extent as the control TRV-gus. Although TMV MP most likely is phosphorylated by multiple protein kinases, the lack of detectible changes in the global degree of TMV MP phosphorylation in BAM1-deficient cells suggests that TMV MP is not an efficient substrate for the enzymatic activity of BAM1. On the other hand, that TMV MP is not recognized by BAM2 (Fig. 1) indicates that BAM2 does not act on TMV MP redundantly with BAM1; this notion is consistent with our observations that loss of function of BAM1 affected viral spread and TMV MP movement in plants that were not compromised for BAM2 expression. Taken together, our data suggest that BAM1 is required for the cell-to-cell, but not systemic, viral movement and that this effect is not mediated by the protein kinase activity of BAM1.

## Discussion

The Arabidopsis genome encodes >600 RLKs^33^, which function to control numerous and diverse aspects of the plant life cycle, such as development, hormone perception, self-incompatibility, and disease resistance^33–37^. Although the role of RLKs in plant susceptibility to viruses has been recently suggested^36^, there has not been any direct evidence of RLKs in cell-to-cell movement of plant viruses and viral MPs. Here we report direct interaction between the receptor-like protein kinase BAM1 from *A. thaliana* and *N. benthamiana* and TMV MP. What could be the role of this interaction in the MP function and TMV movement? Our data suggest that the BAM1–TMV MP complexes localize at or in close vicinity of PD as visualized by colocalization with the PD marker, the PDLP5 protein. The interaction was not detected when the proteins were located ectopically, i.e., in the cell nucleus. The largest BAM1 domain, LRR, is not involved in the recognition of TMV MP, which, instead, is mediated by the TM and/or KD domains. Among these, TM most likely plays an important role in the specificity of BAM1–TMV MP sorting to PD because TM-KD–TMV MP complexes retained their wild-type PD localization pattern but KD–TMV MP complexes were compromised for PD localization. Interestingly, this observation suggests that, in the TM-KD–TMV MP complex, the long since known ability of TMV MP to target to PD^8,38^ is insufficient to promote PD localization. On the other hand, TM-KD—which not only interacted with TMV MP but also exhibited the autophosphorylation activity expected of the BAM1 receptor kinase—failed to phosphorylate TMV MP. Thus, TMV MP does not represent the enzymatic substrate for BAM1, suggesting that this activity is not involved in the potential effect of BAM1 on TMV MP.

The specific PD association of the BAM1–TMV MP complexes suggests their involvement in cell-to-cell movement through the PD channels. We addressed this possibility by analyzing intercellular movement of an isolated TMV MP as well as TMV virions in BAM1 loss-of-function and gain-of-function plants. Two plant species were used, *A. thaliana* and *N. benthamiana*. Because the *N. benthamiana BAM1* gene has not been reported, we cloned it and demonstrated that its protein product, NbBAM1, indeed interacts with TMV MP at PD. In these reverse genetic experiments, the movement of TMV MP was examined between adjacent plant cells, whereas the movement of TMV was examined both locally, i.e., from cell to cell, and systemically, i.e., from leaf to leaf. These analyses uncovered interesting ability of BAM1 to facilitate early stages of viral intercellular spread. Specifically, we observed that BAM1 loss-of-function plants, both Arabidopsis and *N. benthamiana*, were substantially compromised in their ability to support TMV infection. BAM1 gain-of-function Arabidopsis plants, on the other hand, were “super susceptible” to infection, exhibiting much higher virus levels than the control plants. Importantly, these effects of altered cellular amounts of BAM1 on TMV movement occurred during the relatively early stages of the infection process, whereas, at the late stages, the infection returned to the wild-type levels, consistent with BAM1 acting to increase the efficiency of TMV movement. How does BAM1 exert this effect? The ability of BAM1 to interact with TMV MP suggests that it might affect the major function of MP, i.e., its PD transport between plant cells. We confirmed this idea by showing that TMV MP cell-to-cell transport in BAM1 loss-of-function plants is significantly slower than in the control plants. Thus, association of BAM1 with TMV MP at PD most likely enhances the MP ability to translocate through these channels, which, in turn, increases the rate of the spread of the viral infection. The ability of BAM1 to enhance cell-to-cell transport of TMV MP through PD is consistent with its known ability to promote cell-to-cell spread of RNAi^13^.

## Methods

### Plant material

*N. benthamiana* plants were grown on soil in an environment-controlled chamber at 23 °C under a 16-h light (100 μmol photons m^−2^ s^−1^)/8-h dark cycle. The homozygous *A. thaliana* Col-0 *bam1-3* and Ler-0 *bam2-3* mutants were described previously^26^. The homozygous *BAM1* overexpression line (*35* *S:BAM1*) was kindly provided by Dr. Rosa Lozano-Durán (Shanghai Center for Plant Stress Biology, Chinese Academy of Sciences)^13^. The Arabidopsis plants were grown on soil in an environment-controlled chamber at 23 °C under a 12-h light (100 μmol photons m^−2^ s^−1^)/12-h dark cycle. Under these conditions, we observed no differences in the growth rate between any of the plant lines. The mutant and overexpression lines were confirmed by PCR genotyping using the T-DNA-specific primers and gene-specific primers as described^11,13^.

### BiFC assay for protein interactions

The TMV MP and Arabidopsis PAPK1 coding sequences were amplified by PCR using pfu ultra II DNA polymerase (#600674, Agilent), cloned into pDONR207 (#12213013, Invitrogen) by the BP reaction using Gateway BP Clonase II (#11789100, Invitrogen), and transferred into the destination vector pGTQL1221YC (#61705, Addgene) by the LR reaction using Gateway LR Clonase II (#11791020, Invitrogen), resulting in a TMV MP-cYFP construct, encoding the C-terminal portion of YFP (cYFP, amino acids 175–239) fused to the C-terminus of TMV MP^39^. The BAM1-nYFP and BAM3-nYFP constructs in pGTQL1211YN (#61704, Addgene), which encode the N-terminal portion of YFP (nYFP, amino acids 1–174) fused to the C-terminus of BAM1 or BAM3, respectively, were kindly provided by Dr. Rosa Lozano-Durán (Shanghai Center for Plant Stress Biology, Chinese Academy of Sciences)^13^. The Arabidopsis proteins FIE and MEA, known to interact with each other^20^, were expressed from the constructs pNO5 (nYFP-AtFIE) and pNO6 (cYFP-AtMEA) as described^40^. DNA constructs expressing the fusion proteins PDLP5-CFP, PIP2A-CFP, or free CFP that served as PD marker, plasma membrane marker, and nucleocytoplasmic marker, respectively, were described previously^41,42^.

Agroinfiltration into *N. benthamiana* was conducted as described^43^. Two days after infiltration, the infiltrated area was excised and observed under a laser scanning confocal microscope (LSM 900, Zeiss) with a ×40 objective lens and CFP and YFP filters.

### Y2H assays for protein interactions

The coding sequences of BAM1, BAM2, or BAM3 from the pGTQL1211YN vector were transferred to pDONR207 by the BP reaction. For the split ubiquitin Y2H assay, the coding sequences of BAM1, BAM2, and BAM3 and PAPK1 or TMV proteins MP, CP, and RdRp domains were transferred by the LR reaction from pDONR207 into pMetYC-Dest (#105081, Addgene) and pXN22-Dest (#105085, Addgene), respectively. The resulting constructs, expressing BAM1 fused to the C-terminal half (amino acids 35–76) of ubiquitin (Cub), or PAPK1, TMV MP, TMV CP, or TMV RdRP domain fused to the N-terminal half (amino acids 1–34) of the ubiquitin I13G mutant (NubG) were co-transformed into the yeast strain L40 as described^44^, and co-transformants were selected on the synthetic defined (SD) medium deficient for leucine and tryptophan. Protein interactions were detected by histidine prototrophy on the SD medium deficient for leucine, tryptophan, and histidine and supplemented with 10 mM 3-amino-1, 2,4-triazole (3AT) and 0.3 mM methionine to induce the expression of BAM1-Cub, BAM2-Cub, or BAM3-Cub^45^. The N-terminal half of the wild-type ubiquitin (NubWT) in pNubWtXgate (#105080, Addgene) able to self-assemble with any Cub-fused proteins was used as positive control^46^.

For the conventional Y2H assay, the coding sequences of BAM1, BAM2, or BAM3 and TMV MP were transferred by the LR reaction from pDONR207 into pGADT7-GW (#61702, Addgene) and pGBKT7-GW (#61703, Addgene), respectively, and co-transformed into the Y190 cells as described^39^, and co-transformants were selected on the SD medium lacking tryptophan and leucine. Protein interactions were selected by histidine prototrophy on the SD medium deficient for tryptophan, leucine, and histidine and supplemented with 10 mM 3AT. The simian virus 40 large T-antigen (T) expressed from pGADT7-T (#PVT4021, Life Science Market) and murine P53 protein (p53) expressed from pGBKT7-53 (#PVT4019, Life Science Market) were used as a positive control, whereas human Lamin C (LAM) expressed from pGBKT7-lam (#PVT4020, Life Science Market) was used for negative control.

### Purification of recombinant proteins from *E. coli*

The coding sequence of TMV MP or KD was cloned by the LR reaction into a pET28-based Gateway vector, in which the 6xHistidine tag (6xHis) is fused to the N-terminus of the recombinant MP or KD. The resulting pET28 6xHis-MP or pET28 6xHis-KD construct was transformed into *E. coli* strain BL21(DE3) and expressed as described^7^ with minor modifications. Briefly, the bacteria cells, carrying the indicated expression vectors, were grown at 37 °C to optical density at 600 nm (OD_600_) = 0.8–1.0. Protein expression was then induced by adding isopropyl β-d-1-thiogalactopyranoside (IPTG) at final concentration of 1 mM followed by additional cell growth for 3 h at 37 °C. The cells were collected by centrifugation at 4000 rpm for 5 min at 4 °C and lysed by sonication (6 passes, with 10-s sonications and 20-s intervals), and centrifuged at 13,000 rpm for 5 min at 4 °C. For isolation of 6xHis-MP, the pellet was dissolved in denaturation buffer [10 mM Tris pH 8.0, 1 M NaCl, 4 M urea, 1 mM dithiothreitol (DTT), and 1 mM phenylmethylsulfonyl fluoride (PMSF)]. For isolation of 6xHis-KD, the supernatant was collected. The proteins were purified using Ni-NTA resin (Qiagen) as described by the manufacturer. The eluted TMV MP protein was dialyzed against buffer L (10 mM Tris, pH 8.0, 200 mM NaCl, 1 mM EDTA, 10% glycerol, 1 mM DTT, and 1 mM PMSF) for 16 h at 4 °C. The KD was eluted with buffer L sans glycerol, and the eluted protein solution was supplemented with glycerol at 10% (v/v). The eluted proteins were stored at −20 °C before use.

### Transphosphorylation and in vitro phosphorylation assays

The transphosphorylation assay in *E. coli*, which is based on the coexpression of a kinase and its potential substrate, was performed as described^24,25^ with several modifications. First, a DNA sequence encoding GST fused to the N terminus of TMV MP (GST-MP) was produced by overlap PCR; then, coding sequences of BAM1, TM-KD, or KD were joined to GST-MP by a linker, containing a T7 promoter, *lac* operator, and ribosome-binding site from pETDuet-1 (#71146-3, Novagen). These constructs were cloned into pDONR207 by the BP reactions and then transferred into a pET28-based destination vector by the LR reactions. The resulting dual expression plasmids pET28 Flag-BAM1/GST-MP-6xHis, pET28 Flag-TM-KD/GST-MP-6xHis, or pET28 Flag-KD/GST-MP-6xHis were introduced into *E. coli* strain Lemo21 (NEB) and grown at 37 °C until OD_600_ = 0.5. Protein coexpression was induced with 1 mM IPTG at 16 °C for 16 h, and bacterial culture aliquots (1 ml, OD_600_ = 0.5) were collected by centrifugation (13,000 rpm for 1 min) and boiled in 100 µl of sodium dodecyl sulfate-polyacrylamide gel electrophoresis (SDS-PAGE) 1× loading buffer. These samples (20 µl) were resolved on a 12% SDS-PAGE gel and stained with the Pro-Q diamond phosphoprotein gel stain (#P33301, Invitrogen) as described by the manufacturer. Expression of BAM1, TM-KD, and KD or of GST-MP was confirmed by western blotting using anti-Flag M2 antibody (#F3165-.2MG, Sigma) or anti-His-tag antibody (#A00174, Genscript), respectively.

In vitro phosphorylation assay was performed as described^47^ with minor modifications. Briefly, 1 µg of TMV MP and 0.4 µg of KD were assembled in a kinase reaction buffer containing 50 mM HEPES (pH 7.5), 10 mM MgCl_2_, 2 mM DTT, 2 mM EGTA, and 2.2 mM CaCl_2_ and kept on ice until ATP was added to a final concentration of 100 mM. The reaction mixture was immediately transferred to 30 °C and incubated for 20 min. The reaction was stopped by adding 1 volume of SDS-PAGE 2× loading buffer and boiling for 5 min. The 20-µl aliquots of the reaction mixtures were electrophoretically resolved on a 12% SDS-PAGE gel, followed by staining with the Pro-Q diamond phosphoprotein gel stain. The total protein in the phosphostained gel was detected by subsequent staining with Coomassie brilliant blue. Protein band images were recorded using a Gel Doc XR+ gel documentation system (#170,8185, Bio-Rad).

### Virus maintenance and inoculation

TMV and its TRBO-CP-G variant were maintained in *N. benthamiana* plants. To purify the virus-containing sap, ca. 50 mg of the infected leaves was ground in liquid nitrogen and suspended in 1 ml of ice-cold phosphate buffer (0.05 M, pH 7.4); supernatant was collected by centrifugation at 15,000 × *g* and diluted four times in the same phosphate buffer. For plant inoculation, two leaves of 2-week-old Arabidopsis or 3-week-old *N. benthamiana* plant (from sowing) were inoculated with 20 or 100 µl of the diluted sap, respectively.

### Virus-induced gene silencing

To silence expression of *NbBAM1*, a 0.7-kb fragment of *NbBAM1* was amplified and cloned in reverse complementary orientation into the restriction endonuclease site *Mlu*I of the pTRV binary vector. For silencing controls, 0.7-kb fragments of *GusA* (NCBI accession number S69414) and *NbPDS* (NCBI accession EU165355) were amplified and cloned into the *Mlu*I site of pTRV. *GusA* was used as negative control because it has no sequence homologs in the *N. benthamiana* genome (Sol Genomics Network, *N. benthamiana* genome version 1.0.1, predicted cDNAs), whereas *NbPDS* was used as positive control because the photobleached phenotype caused by silencing of the endogenous *PDS* gene is easily detectable. The 0.7-kb length of the target sequence used for silencing represents the optimal insert size that reduces TRV virulence to prevent damage to plants while maintaining the silencing efficacy^48^. The TRV constructs were transformed into the Agrobacterium strain GV2260. For agroinfiltration, the overnight cultures of the Agrobacterium were harvested by centrifugation and suspended in the MMA buffer (10 mM MgCl_2_, 10 mM MES, 200 µM acetosyringone) to OD_600_ of 0.1. Suspension of bacterial cells harboring constructs for TRV RNA2s (pTRV2) was mixed in 1:1 ratio, one by one, with bacteria harboring the TRV RNA1 construct. Each combination mixture was separately infiltrated in two abaxial sides of two largest mature leaves of 2-week-old *N. benthamiana*, with three plants per experiment. Two weeks after infiltration, when the white bleached phenotype developed in systemic leaves of the positive control plants, the corresponding leaves of negative control and experimental plants were harvested. Expression levels of *NbBAM1* were accessed by RT-qPCR as described below.

### Movement assays of TMV MP and TMV virions

For TMV MP cell-to-cell movement assay, the coding sequence of MP was transferred by Gateway cloning from pDONR207 into pPZP-RCS2A-DEST-ECFP-N1, producing a binary construct encoding the TMV MP fusion to N-terminus of CFP, and transformed it into the Agrobacterium strain EHA105 as described^49^, followed by agroinfiltration as described above with OD_600_ of Agrobacterium cell suspension ranging from 10^−^^4^ to 10^−3^; these conditions were calibrated to optimize expression in single transformed cells (Fig. S5). Two and three days later, the infiltrated leaves were harvested and observed using a laser scanning confocal microscope with ×10 and ×40 objective lenses and CFP filter. Single cells (indicating no cell-to-cell movement) and clusters of two and three cells containing the CFP signal (indicating cell-to-cell movement) were scored in three different leaves from three different plants.

For TMV cell-to-cell movement assay, a construct encoding TMV-GFP without CP (pTRBO-G, #80083, Addgene)^29^ was transformed into Agrobacterium EHA105. Agroinfiltration was used to introduce pTRBO-G into *N. benthamiana* leaves as described above with the concentration of the bacterial suspension at OD_600_ ranging from 10^−5^ to 10^−4^. Two and three days after agroinfiltration, the infiltrated leaves were sectioned and observed using a laser scanning confocal microscope with a ×10 objective lens and GFP filter. Diameters of 30 GFP loci per experiment were measured using the ImageJ software (https://imagej.nih.gov/ij/) and statistically evaluated as described above.

For long-distance movement of TMV, a modified TRBO-G was constructed by replacing a part of TMV 3’ untranslated region (UTR) with a fusion construct containing the CP cistron of TMV strain U5. Briefly, a PCR product containing the 3’ UTR of TMV (nucleotides from position 6192 to 6365, NCBI accession number V01408) and the CP-encoding cistron from TMV-strain U5 (nucleotides from position 5498 to 6502, NCBI accession MH730970) was amplified from p30B^50^. Next, using the overlap PCR, this fragment was fused to the second fragment containing a hammer head ribozyme site and a 35S terminator amplified from pTRBO-G (nucleotides from position 6724 to 7431), and the resulting fusion product was introduced into the *Not*I/*Pme*I restriction endonuclease sites of pTRBO-G. This final construct, designated pTRBO-CP-G, was transformed into Agrobacterium EHA105 and agroinfiltrated into *N. benthamiana* as described above with the concentration of the bacterial suspension at OD_600_ ranging from 10^−6^ to 10^−3^; this low bacterial culture density was chosen to allow infection in separate foci. Ten days after agroinfiltration, the infiltrated and upper leaves from the infected plants were detached and photographed under a long-wave ultraviolet lamp using a digital camera (Sony).

### Quantitative RT-PCR

To quantify *BAM1* expression or TMV RNA levels in Arabidopsis, about 50 mg of upper, non-inoculated leaves were harvested at 6 and 13 dpi. Total RNA was extracted by Triazol (#15596026, Invitrogen) according to the manufacturer’s instructions and utilized as template to synthesize cDNA using the RevertAid Revert Transcription Kit (#K1691,Thermofisher) and hexa-random primers. Quantitative PCR (qPCR) was performed using the EvaGreen Dye protocol as recommended by the manufacturer (#31000, Gold Biotechnology) in a MiniOpticon real-time PCR system (#CFB-3120, Bio-Rad). Arabidopsis *ACTIN 2* (AT3G18780) was used as an internal control for normalization. To quantify *NbBAM1* expression in the TRV-silenced *N. benthamiana* plants, we utilized the same protocol, except that the RNA was derived from 100 mg samples of the upper, non-inoculated leaves collected at day 14 after TRV treatment. *N. benthamiana PP2A* was used as an internal control for normalization^51^. Primers used for these qPCR reactions are listed in Table S1. Fold change was calculated by delta-delta Ct method as described^52^.

### Statistics and reproducibility

All representative images reflect a minimum of three biological replicates. The quantitative data in Figs. 1 and 3–5 were derived from at least three biological replicate experiments. The exact number of biological replicates performed per experiment are indicated in the figure legends. Statistical significance of differences in sample means was evaluated by one-tailed *t* test using the Excel 2019 (Microsoft) software, with *P* values <0.05, 0.01, or 0.001 corresponding to the statistical probability of >95, 99, or 99.9%, respectively, considered statistically significant.

### GenBank accession number

The NCBI accession number for the *NbBAM1* sequence reported in this paper is MT623393.

### Reporting summary

Further information on research design is available in the Nature Research Reporting Summary linked to this article.

## Supplementary information

Supplementary Information

Reporting Summary

## Data Availability

Source data underlying plots shown in Figs. 1 and 3–5 are available in Supplementary Tables S2–S5. These data sets contain the raw data of quantifications, including co-localization, fold changes in relative level of genes and TMV genomic RNA, and measurements of cell-to-cell movements of protein TMV MP and virus TMV GFP. In addition, all relevant data are available from the corresponding authors upon request.
