# Supplementary material for: Receptor-like kinase BAM1 facilitates early movement of the Tobacco mosaic virus
Source: Commun Biol. 2021 Apr 30;4:511. doi: 10.1038/s42003-021-02041-0 (PMC8087827; doi:10.1038/s42003-021-02041-0)
Supplement: Supplementary file 1 — Supplementary Information [file 42003_2021_2041_MOESM1_ESM.pdf]

Supplementary Information

Supplementary Methods

Cloning of RdRp domains and CP for the BiFC and Y2H assays

TMV RdRp is large and contains four main domains [1], methyltransferase (MT), nonconserved region I (NSI), nonconserved II (NSII), and helicase (HL). Coding sequences of each of these RdRP domains and CP were amplified from pTRBO-G (#80083, Addgene) using pfu ultra II DNA polymerase (#600674, Agilent) and primers listed in Table S1, and cloned into pDONR207 (#12213013, Invitrogen) by the BP reaction using Gateway BP Clonase II (#11789100, Invitrogen). These entry clones in pDONR207 were transferred to the corresponding destination vectors for use in the BiFC and Y2H assays by the LR reaction using Gateway LR Clonase II (#11791020, Invitrogen).

Construction of BAM1 mutants for the BiFC assay

DNA sequences of BAM1 coding for amino acid residues 1 to 622 (LRR), 623 to 1003 (TM-KD), and 678 to 974 (KD) were amplified using pfu ultra II DNA polymerase (#600674, Agilent) and primers listed in Table S1. The PCR products were cloned into pDONR207 (#12213013, Invitrogen) by the BP reaction using Gateway BP Clonase II (#11789100, Invitrogen) and transferred into the destination vector pGTQL1211YN (#61704, Addgene) by the LR reaction using Gateway LR Clonase II (#11791020, Invitrogen).

Cell-free phosphorylation assays

Cell wall-enriched fractions was purified from 2 g of fully-expanded leaves of *N.*

benthamiana 2 weeks after TRV inoculation, when the highest silencing effect is observed, as described [2], resuspended in 2 ml of buffer H (0.1M HEPES pH 7.4, 10 mM EDTA, 5 mM DTT, 1 mM PMSF), and used in the cell-free phosphorylation assay of TMV MP as described [2]. Briefly, 25-µl aliquots of cell wall fraction preparation, containing ca. 10 µg protein in buffer H, were mixed with 5 µl 10x kinase buffer (0.2 M HEPES pH 7.4, 50 mM $MgCl_2$, 0.5 M KCl) and 10 µl buffer H containing 1.0 µg of purified TMV MP, and the reactions were initiated by addition of 10 µl 100 mM ATP, followed by incubation for 15 min at 25°C. The reaction was stopped by addition of 50 µl SDS-polyacrylamide gel electrophoresis (SDS-PAGE) 2X loading buffer and boiling for 5 min. The 20-µl aliquots of the reaction mixtures were resolved by SDS-PAGE and, followed by staining with the Pro-Q diamond phosphoprotein gel stain (#P33301, Invitrogen) as described by the manufacturer. The total protein in the phosphostained gel was detected by subsequent staining with Coomassie brilliant blue. Protein band images were recorded using a Gel Doc XR+ gel documentation system (#170,8185, Bio-Rad).

Table S1. Primers used for DNA amplification, molecular cloning, and detection

No.	Primer name	Sequence (5′ to 3′)	Purpose
1	attB1-PAPK1 Fw	ggggacaagtttgtacaaaaaagcaggctcaatgg acttgaaaatggataatgttattg	Cloning of papk1 from *arabidopsis thaliana*
2	attB2-PAPK1 Rv	ggggaccactttgtacaagaaagctgggtgtttgcg gatcgaaagaagctc	As above
3	attB1-TMV MP Fw	ggggacaagtttgtacaaaaaagcaggctcaatgg ctctagttgttaaaggaaaagtg	Cloning of tmv mp
4	attB2-TMV MP Rv	ggggaccactttgtacaagaaagctgggtgaaacg aatccgattcggcga	As above
5	attB1-TMV MT Fw	ggggacaagtttgtacaaaaaagca ggctcaatggcatacacacagacagctac	Cloning of TMV RdRP methyl transferase domain
6	attB2-TMV MT Rv	ggggaccactttgtacaagaaagctgg gtggtaaacctctctattagaggccgg	As above
7	attB1-TMV NSI Fw	ggggacaagtttgtacaaaaaagca ggctcaatgaaggagtttttagtcaccaga	Cloning of TMV RdRP non-structure domain 1
8	attB2-TMV NSI Rv	ggggaccactttgtacaagaaagctgg gtgggacaaggattgtaacaaagatttgt	As above
9	attB1-TMV NSII Fw	ggggacaagtttgtacaaaaaagca ggctcaatgacgttttacctgcatactaagc	Cloning of TMV RdRP non-structure domain 2
10	attB2-TMV NSII Rv	ggggaccactttgtacaagaaagctgg gtggtcggaataaacaacagactcagagc	As above
11	attB1-TMV HL Fw	ggggacaagtttgtacaaaaaagca ggctcaatggcgaaactcagaactctgcg	Cloning of TMV RdRP helicase domain
12	attB2-TMV HL Rv	ggggaccactttgtacaagaaagctgg gtgttgtgttcctgcatcgaccttatac	As above
13	attB1-TMV CP Fw	ggggacaagtttgtacaaaaaagcaggctcaatgt cttacagtatcactactccatc	Cloning of TMV CP
14	attB2-TMV CP Rv	ggggaccactttgtacaagaaagctgggtgagttgc aggaccagaggtcc	As above
15	attB1-PDLP5 Fw	ggggacaagtttgtacaaaaaagcaggctcaatga tcaagacaaagacgacgtc	Cloning of PDLP5 from *A. thaliana*

16	attB2-PDLP5 Rv	ggggaccactttgtacaagaaagctgggtgtttacaccatttctcatcttgtaattttc	As above
17	attB1-PIP2A Fw	ggggacaagtttgtacaaaaaagca ggctcaatggcaaaggatgtggaagc	Cloning of PIP2A from *A. thaliana*
18	attB2-PIP2A Rv	ggggaccactttgtacaagaaagctgg gtggacgttggcagcacttctga	As above
19	AttL1	tcgcgttaacgctagcatggatctc	Confirmative PCR and sequencing of entry clones
20	AttL2	gtaacatcagagattttgagacac	As above
21	BAM1 1436 Fw	ccggcgttcagaaacttcttc	Sequencing of BAM1
22	BAM1 1511 Rv	tgctgaagcttccctacctc	As above
23	AttB1 Fw	ggggacaagtttgtac aaaaaagcaggct	Confirmative PCR and sequencing of destination clones
24	AttB2 Rv	ggggaccactttgta caagaaagctgggt	As above
25	35S Promoter Fw	ctatccttcgcaagacccttc	Confirmative PCR of destination binary vector
26	GFP 86 Rv	ctgaacttgtggccgtttacgt	Confirmative PCR of BiFC vector with nYFP
27	GFP 604 Rv	ggtagtggttgtcgggcag	Confirmative PCR of BiFC vector with cYFP
28	BAM1 5'S	gtctaacaacatgttcaccgg	Genotyping PCR of Arabidopsis *bam1-3* mutant line, gene-specific
29	BAM1 3'I	gagagctcgtttctgctcagatca	As above
30	LBa1	atggttcacgtagtgggccatc	Genotyping PCR of Arabidopsis *bam1-3* mutant line, Ti-DNA-specific
31	Actin2 1093 Fw	gtggtcgtacaaccggtatt	Reference for RT-qPCR of transcripts in Arabidopsis, span exon-exon boundary to avoid genomic DNA contamination

32	Actin2 1288 Rv	cacgtccagcaaggtcaaga	Reference for RT-qPCR of transcripts in Arabidopsis
33	BAM1 2583 Fw	cggctacatagctccagagtat	RT-qPCR of BAM1 transcripts in *A. thaliana*, span exon-exon boundary to avoid genomic DNA contamination
34	BAM1 2681 Rv	cttccggtgacgagttccaa	RT-qPCR of BAM1 transcripts in *A. thaliana*, specific for BAM1 but not for BAM2 or BAM3
35	AttB1 NbBAM1 Fw	ggggacaagtttgtacaaaaaagcaggctcaatgc gtcttcttcttccccttattc	Cloning of *NbBAM1* from *Nicotiana benthamiana*
36	AttB2 NbBAM1 Rv	ggggaccactttgtacaagaaagctgggtggatgct gagtaggtcaggtggag	As above
37	NbBAM1 1456 Fw	gggaacaaattttcggggcg	Sequencing of *NbBAM1*
38	NbBAM1 1556 Rv	ggcacaatgggtccagagaa	As above
39	NbBAM1 989 MluI Fw	gcacgcgtgggacttgcctgagctagag	Cloning a 0.7 kb fragment of *NbBAM1* into pTRV2
40	NbBAM1 1704 MluI Rv	gcacgcgtaggggcaggaatactcccaa	As above
41	GusA 969 MluI Fw	gcacgcgtcaactcctaccgtacctcgc	Cloning a 0.7 kb fragment of *GusA* into pTRV2, used as negative control
42	GusA 1668 MluI Rv	gcacgcgtggtcgcaaaatcggcgaaat	As above
43	NbPDS 1005 MluI Fw	cgacgcgtgagtcaaaaggtggccaagtca	Cloning a 0.7 kb fragment of PDS from *N. benthamiana* into pTRV2, used as positive control
44	NbPDS 1726 MluI Rv	cgacgcgtacatcttctggctccggccaag	As above
45	NbPP2A 1690 Fw	gaccctgatgttgatgttcgct	Reference for RT-qPCR of transcripts in *N. benthamiana*
46	NbPP2A 1812 Rv	gagggatttgaagagagatttc	As above
47	NbBAM1 1767 Fw	ccgggtactgggcagtttag	RT-qPCR of BAM1 transcripts in *N. benthamiana*
48	NbBAM1 1901 Rv	aaagctcctcgttggtgagg	As above

49	TMV MP 552 Fw	agggcccatggaacttacag	RT-qPCR of TMV RNA, specific for TMV MP
50	TMV MP 670 Rv	tccctttgcggacatcactc	As above
51	TrfA 987 Fw	tacccgctcaagctggaaac	Construction of pTRBO-CP-G
52	TMGMV-Hmz Fw	cggcccacctaggcggatgtgttttccgggctga	As above
53	TMGMV-Hmz Rv	acatccgcctaggtgggccgctacccgcggttag	As above
54	TMV 6192 NotI Fw	aaactagggcggccgcggtagtcaagatgcataat aaataacgg	As above
55	AttB2 LRR Rv	ggggaccactttgtacaagaaagctgggtggtcgac taccgtctttacaaggaccaag	Used together with primer no. 1 for amplification of LRR from amino acid 1 to 622
56	AttB1 TM-KD Fw	ggggacaagtttgtacaaaaaagcaggctcaatgg ttgctaaaggaggtcaccagag	Used together with primer no. 2 for amplification of TM-KD from amino acid 623 to 1003
57	BAM1-stop-spacer Rv	gtacaatacgattactttctgttcgatcatagattgag tagatccggcgg	Used together with primer no. 2 for amplification of BAM1 or TM-KD for fusion PCR to linker and GST-MP
58	AttB1 KD Fw	ggggacaagtttgtacaaaaaagcaggctcaacag ctttccagagactagac	Amplification of KD for fusion PCR to linker and GST or GST-MP
59	KD-stop-spacer Rv	gtacaatacgattactttctgttcgatcacgacggtg gcaacttcgggat	As above
60	pDUET spacer Fw	tcgaacagaaagtaatcgtattgtac	Amplification of linker for fusion PCRs to generate dual expression constructs
61	pDUET spacer Rv	catatgtatatctccttcttatacttaac	As above
62	Spacer-GST Fw	gttaagtataagaaggagatatacatatgtcccta tactaggttattgg	Amplify GST for fusion PCRs to generate dual expression constructs
63	Factor Xa site Rv	acgaccttcgatcagatccga	As above
64	Xa-TMV MP Fw	atcggatctgatcgaaggtcgtgctctagttgttaaa ggaaaagtg	Used together with primer no. 4 for amplification TMV MP for fusion of GST and MP

Table S2. Pearson's correlation coefficients of YFP and CFP signals generated from 30 regions of interest (ROI) using ImageJ software

No.	PDLP5	PIP2A	Free CFP
1	0.59	0.46	0.03
2	0.53	0.51	0.02
3	0.67	0.7	0.11
4	0.71	0.54	0.14
5	0.67	0.69	0
6	0.61	0.71	-0.01
7	0.74	0.61	0.24
8	0.73	0.55	0.3
9	0.76	0.44	0
10	0.63	0.48	0.03
11	0.77	0.54	0
12	0.87	0.46	0.06
13	0.77	0.61	0
14	0.7	0.65	-0.09
15	0.8	0.44	0.1
16	0.79	0.51	0
17	0.69	0.7	0
18	0.72	0.7	0
19	0.75	0.71	0.3
20	0.73	0.77	-0.14
21	0.7	0.87	0
22	0.71	0.83	0.28
23	0.67	0.64	0
24	0.75	0.53	0.37
25	0.7	0.56	0.3
26	0.77	0.63	0.14
27	0.73	0.44	0.04
28	0.81	0.48	0.06
29	0.84	0.42	0.39
30	0.71	0.52	0.14

Table S3. Relative expression levels of *BAM1* (**a**) and relative levels of TMV genomic RNA (**b**) in systemic leaves of the wild type, the gain-of-function (*35S:BAM1*), and loss-of-function *Arabidopsis* Col-0 lines (*bam1-3*) 6 and 13 days post inoculation.

a

	Expression level at 6dpi			Expression level at 13dpi		
No.	WT	*35S:BAM1*	*bam1-3*	WT	*35S:BAM1*	*bam1-3*
1	1.10	58.48	0.09	0.69	0.983417	0.190732
2	0.725426	1.968922401	0.096254856	1.798427	1.777006	0.299598
3	1.360656	3.17213991	0.044828963	1.559648	3.56088	0.421899
4	1.11751	1.889143299	0.131077587	2.251947	2.780348	0.351781
5	0.624192	2.968018611	0.055891954	1.097857	3.307641	0.245459
6	1.320969	5.05116414	0.046649736	0.442347	1.357674	0.511733

b

	TMV level at 6dpi			TMV level at 13dpi		
No.	WT	*35S:BAM1*	*bam1-3*	WT	*35S:BAM1*	*bam1-3*
1	0.396611	0.09906406	0.151186956	994.9718	949.8765	444.4098
2	0.035518	35.46663762	0.095352291	587.9482	581.6253	114.7064
3	0.823259	0.099124295	0.06402059	1495.011	1277.98	266.1414
4	8.330317	459.0470316	0.060610337	450.5282	916.6367	462.1419
5	9.264448	0.459548291	0.184247063	1360.622	159.5464	503.6862
6	1.117296	0.088766912	0.21091557	1418.675	505.9933	522.2082

Table S4. Relative expression levels of *BAM2* (a) and relative levels of TMV genomic RNA (b) in systemic leaves of the wild type and loss-of-function *Arabidopsis* Ler-0 line (*bam2-3*) 6 days post inoculation.

a

	Expression levels	
No.	Ler WT	Ler bam2
1	0.490112	0.10583
2	1.46768	0.13282
3	1.080487	0.095444
4	1.038265	0.184844
5	1.239212	0.120335

b

	TMV levels	
No.	Ler WT	Ler bam2
1	0.00082	0.000139
2	0.000429	0.00027
3	1.604181	0.000373
4	0.001	0.000517
5	3.393297	2.158627

Table S5. Effect of VIGS of *NbBAM1* on cell-to-cell movement of TMV MP. **a** expression levels of *NbBAM1* in *PDS*- and *NbBAM1*-silenced *N. benthamiana* plants 2 weeks after treatment with TRV vectors. **b** Percentage of moved cells (>1 cell / cluster) of TMV MP in *NbBAM1*-silenced *N. benthamiana* plants 60 and 72 hours post inoculation. **c** The extent of MP-CFP cell-to-cell movement was scored as frequency of cell clusters containing the CFP signal and composed of 2 or 3 cells/cluster.

a

	Fold change		
No.	Healthy	TRV-gus	TRV-bam1
1	0.988329	0.55442	0.106108
2	0.884456	0.932088	0.147466
3	1.14399	1.244736	0.119917

b

	Percentage of moved cells (>1 cell / cluster)					
	60 hpi			72 hpi		
No.	1	2	3	1	2	3
Healthy	48.3871	43.33333	51.42857	48.48485	45.45455	59.375
TRV-gus	52.17391	53.84615	45.71429	61.36364	53.48837	50
TRV-bam1	30.43478	36.17021	37.2093	45.94595	48.27586	43.33333

c

		Percentage of cells								
		Healthy			Vector			Silenced		
	No.	1	2	3	1	2	3	1	2	3
60 hpi	2-cell cluster	38.7	30	42.8571	39.1304	38.4615	34.2857	28.2608	34.0425	32.5581
	3-cell cluster	9.67	13.3333	8.57142	13.0434	15.3846	11.4285	2.17391	2.12766	4.65116
72 hpi	2-cell cluster	39.3	39.3939	43.75	40.9090	41.8604	40	40.5405	37.9310	40
	3-cell cluster	9.09	6.06060	15.625	20.4545	11.6279	10	5.40540	10.3448	3.33333

Table S6. Size of infection loci of TMV without (**a**) and with CP (**b**)

a

	Diameter (μm) of GFP expressing infection loci			
	2dpi		3dpi	
No.	TRV-gus	TRV-bam1	TRV-gus	TRV-bam1
1	290	190	350	260
2	280	110	450	400
3	310	170	400	250
4	255	210	420	270
5	260	150	345	255
6	220	100	355	200
7	200	120	340	245
8	275	100	360	195
9	265	195	255	150
10	200	150	245	205
11	240	205	205	150
12	220	155	335	105
13	250	100	310	95
14	285	120	270	110
15	270	115	370	120

b

Diameter of GFP expressing infection loci at 3 dpi								
	Size (mm)			Size (mm)			Size (mm)	
No.	TRV-gus	TRV-bam1	No.	TRV-gus	TRV-bam1	No.	TRV-gus	TRV-bam1
1	0.486	0.555	26	0.519	0.618	51	0.665	0.412
2	0.734	0.335	27	0.617	0.458	52	0.666	0.510
3	0.336	1.266	28	0.814	1.027	53	0.708	0.378
4	0.507	0.320	29	0.990	0.436	54	0.428	0.433
5	0.837	0.830	30	0.777	0.407	55	0.729	0.571
6	0.850	0.795	31	1.028	0.372	56	1.038	0.554
7	0.709	0.636	32	1.061	0.491	57	0.874	0.478
8	0.731	0.331	33	0.744	0.659	58	0.779	0.852
9	0.596	0.495	34	0.695	0.490	59	0.757	0.427
10	0.880	0.600	35	0.718	0.646	60	0.684	0.320
11	0.560	0.447	36	0.849	0.342	61	0.588	0.572
12	0.598	0.463	37	1.168	0.351	62	0.917	0.541
13	0.704	0.863	38	0.767	0.677	63	0.756	0.340
14	0.763	0.549	39	0.651	0.320	64	0.739	0.563
15	0.730	0.710	40	0.892	0.661	65	0.372	0.758
16	0.846	0.387	41	0.650	0.662	66	0.672	0.320
17	0.580	0.346	42	0.895	0.839	67	0.688	0.581
18	0.391	0.310	43	0.582	0.477	68	0.747	0.331
19	0.332	0.344	44	0.786	1.141	69	0.894	0.402
20	0.630	0.410	45	0.881	0.913	70	0.461	0.544
21	0.776	0.810	46	0.794	0.874	71	0.443	0.393
22	0.771	0.378	47	0.774	0.414	72	1.000	0.402
23	0.471	0.351	48	0.800	0.327	73	1.072	0.320
24	0.788	0.412	49	0.649	0.379	74	0.724	0.445
25	0.638	0.465	50	0.720	0.926	75	1.177	0.561

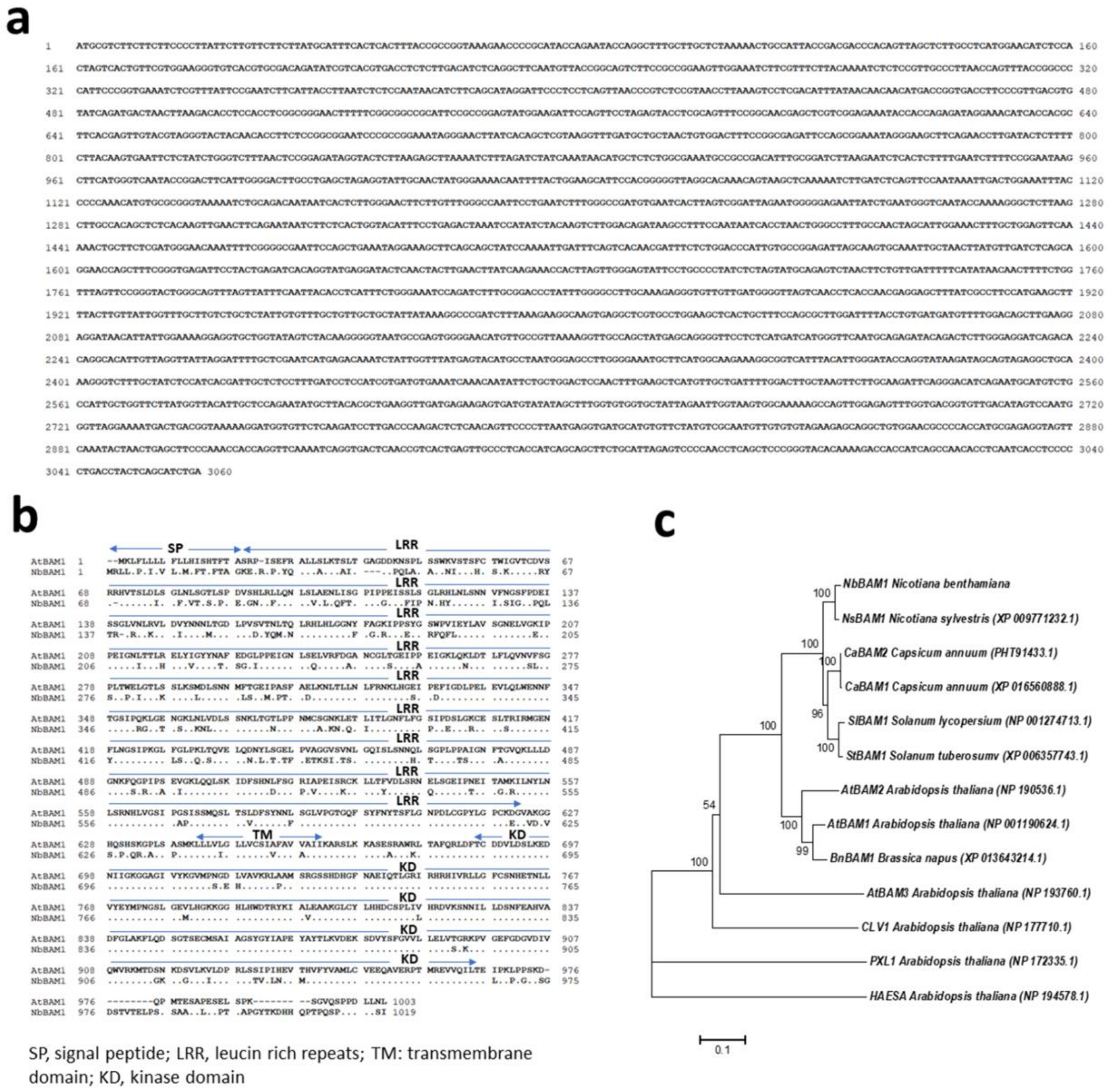

Figure S1. BAM1 homolog of *N. benthamiana*. a Amino acid sequence alignment of BAM1 proteins from *A. thaliana* (AtBAM1) and *N. benthamiana* (NbBAM1). The amino acid sequence of NbBAM1 was aligned with that of AtBAM1 (NP001190624.1) using ClustalX (ver. 2.1) (http://www.clustal.org/clustal2/). Arrows delineate the following indicated domains: SP, signal peptide; LRR, leucine-rich repeats; TM, transmembrane domain; KD, protein kinase domain. Identical amino acids are indicated by periods. **b** Nucleotide sequence of the *NbBAM1* cDNA. **c** Phylogenetic tree of BAM1 homologs from different plant species. NtBAM1 is indicated by asterisk. The evolutionary history was inferred using the Neighbor-Joining method [3]. The optimal tree with the sum of

branch length of 2.3252050 is shown. The percentage of replicate trees in which the associated taxa clustered together in the bootstrap test (1,000 replicates) are shown next to the branches [4]. The tree is drawn to scale, with branch lengths in the same units as those of the evolutionary distances used to infer the phylogenetic tree. The evolutionary distances were computed using the Poisson correction method [5] and are in the units of the number of amino acid substitutions per site. The analysis involved 13 amino acid sequences. All positions containing gaps and missing data were eliminated. There were 940 positions in the final dataset. Evolutionary analyses were conducted using the Molecular Evolutionary Genetics Analysis tool (MEGA, version 6.0.5 for Mac OS) (http://www.megasoftware.net) [6], which also generated this description of the analysis. Scale bar, 0.1 amino acid substitutions per site.

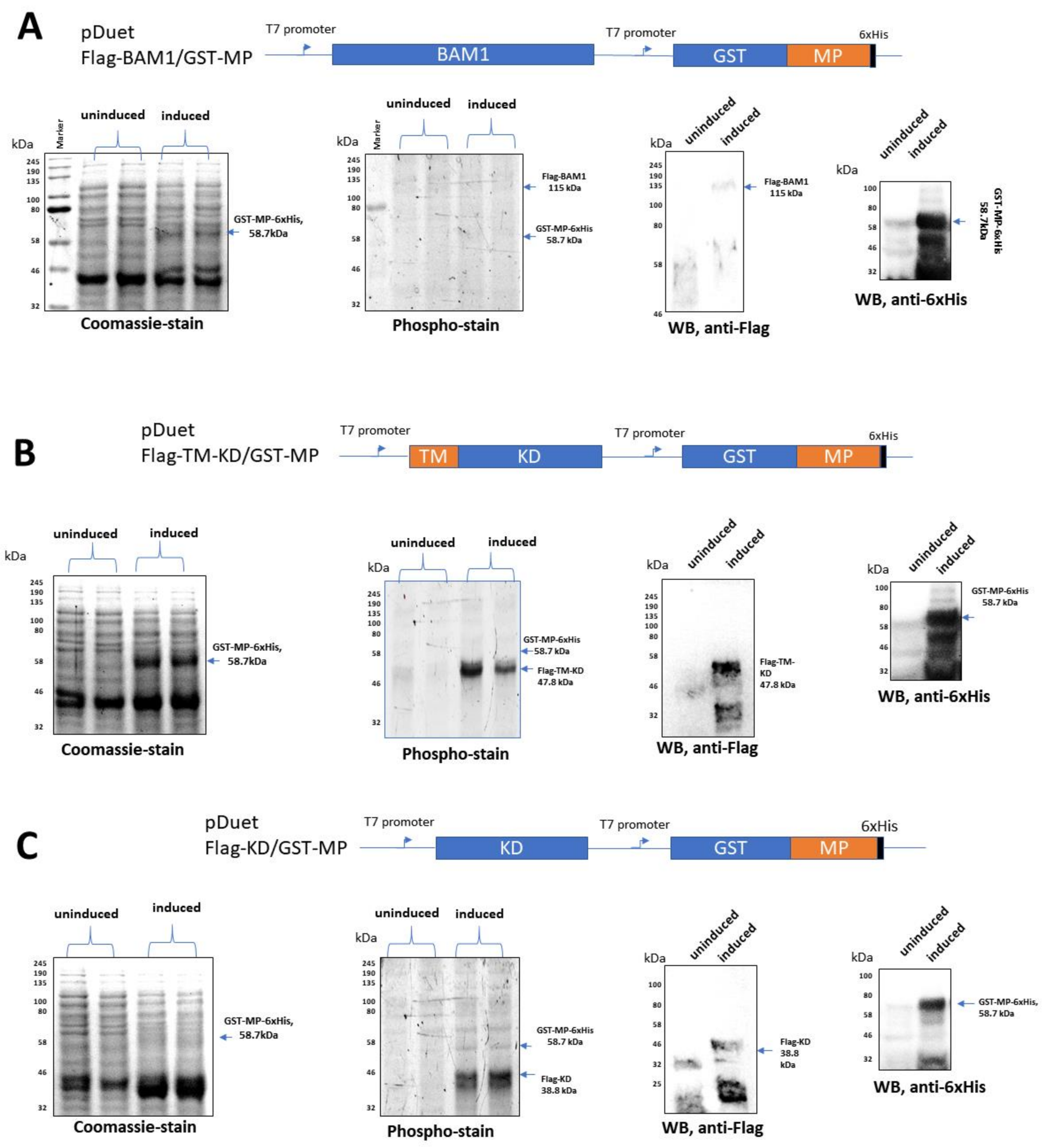

Figure S2. Phosphorylation of GST-MP in *Escherichia coli*. Transphosphorylation assay was conducted under dual expression of (**a)** BAM1 and GST-MP, (**b)** BAM1 TM-KD and GST-MP, or (**c)** BAM1 KD and GST-MP in *E. coli* Lemo21(DE3). After induction, cell pellet was subjected to SDS-PAGE followed by Coomassie staining or phosphoprotein stain staining, or western blotting with anti-Flag or anti-Histidine antibody.

a

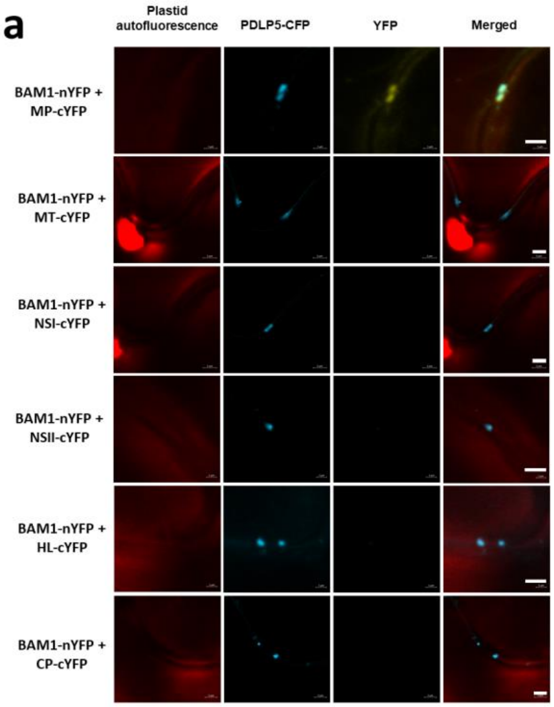

b

Split-ubiquitin Y2H

Combinations	Results
BAM1-Cub + MP-NubG	Positive
BAM1-Cub + MT-NubG	Negative
BAM1-Cub + NSI-NubG	Negative
BAM1-Cub + NSII-NubG	Negative
BAM1-Cub + HL-NubG	Negative
BAM1-Cub + CP-NubG	Negative

Conventional Y2H

Combinations	Results
AD-T + BD-P53	Positive (positive control)
AD-BAM1 + BD-MT	Negative
AD-BAM1 + BD-NSI	Negative
AD-BAM1 + BD-NSII	Negative
AD-BAM1 + BD-HL	Negative
AD-BAM1 + BD-CP	Negative
AD-MT + BD-BAM1	Negative
AD-NSI + BD-BAM1	Negative
AD-NSII + BD-BAM1	Negative
AD-HL + BD-BAM1	Negative
AD-CP + BD-BAM1	Negative

Figure S3. Lack of interaction between BAM1 and TMV RdRp domains or CP in BiFC and split-ubiquitin Y2H. a Protein interaction was analyzed by BiFC in *N. benthamiana* leaves agroinfiltrated with the indicated combinations (1:1 w/w ratio) of the tested expression constructs and the expression construct for PDLP5-CFP as a PD marker. YFP signal is in yellow; CFP signal is in cyan. Images were recorded 48 h after agroinfiltration and are single confocal sections representative of multiple independent experiments (N=20 images from 5 plants). Scale bars = 2 µm. **b** Summarized results of protein interaction analyzed by split-ubiquitin and conventional Y2H assays.

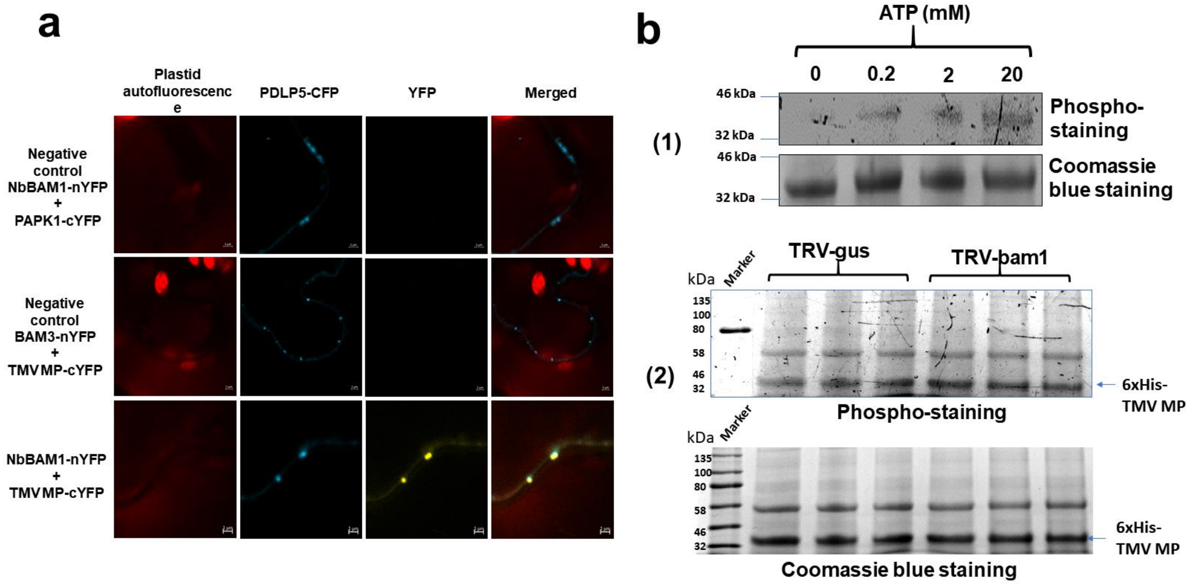

Figure S4. Interaction of NbBAM1 with TMV MP and TMV MP phosphorylation by cell wall-enriched fractions. **a** Interaction. Protein interaction was analyzed by BiFC in *N. benthamiana* leaves agroinfiltrated with the indicated combinations (1:1 w/w ratio) of the tested expression constructs and an expression construct for PDLP5-CFP as a PD marker. YFP signal is in yellow; CFP signal is in cyan. Images were recorded 48 h after agroinfiltration and are single confocal sections; images are representative of multiple independent experiments (N=20 images from 5 plants). Scale bars = 5 µm. **b** Phosphorylation. TMV MP was phosphorylated in a cell-free system using cell wall-enriched fractions of control (TRV-gus) and *NbBAM1*-silenced plants (TRV-bam1). (1) Dose response of TMV MP phosphorylation by TRV-gus to ATP concentration. (2) TMV MP phosphorylation by TRV-gus and TRV-bam1. Triplicate experiments are shown for each condition. The assay was performed in the presence of 20 mM ATP. TMV MP phosphorylation was detected by phosphostaining with the Pro-Q diamond gel stain, and the equal input of TMV MP was verified by Coomassie blue staining.

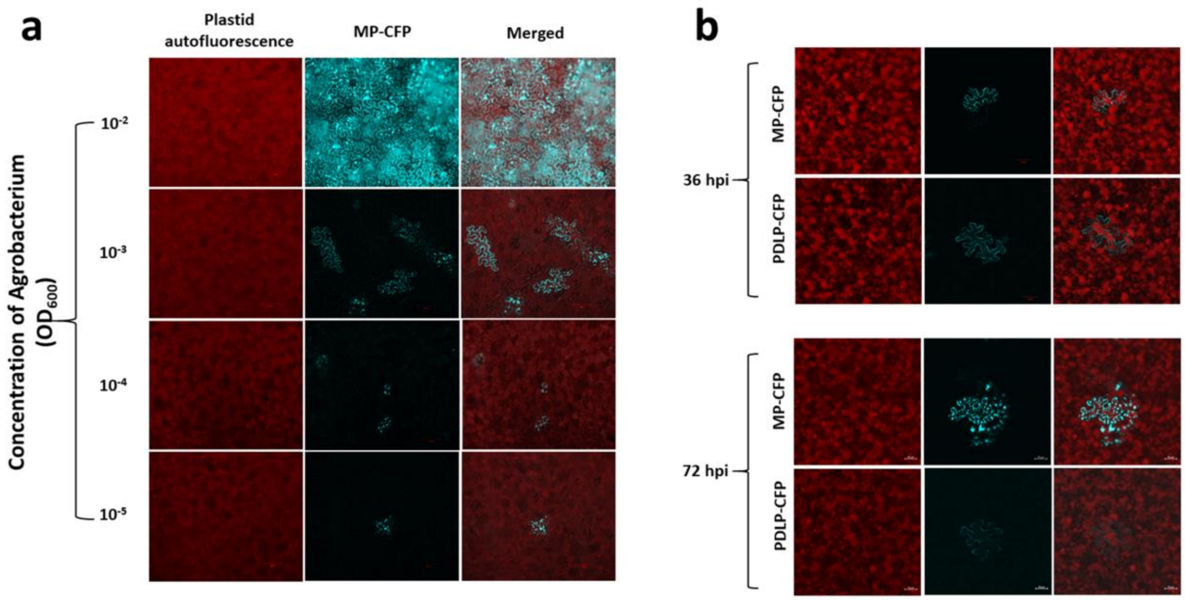

Figure S5. Calibration of bacterial cell density during agroinfiltration to allow MP-CFP expression in single transformed cells. a Confocal microscopic images of *Nicotiana benthamiana* leaves 48 hours post infiltration (hpi) with serially diluted Agrobacterium cell suspensions harboring an MP-CFP-expressing construct. **b** Representative images of single cells (at 36 hpi) and multi-cell clusters (at 72 hpi) that express TMV MP-CFP. PDLP5-CFP was used as negative control that does not move from cell to cell. Images are single confocal sections representative of multiple independent experiments (N=20 images from 5 plants).

Supplementary References

1. Wang LY, Lin SS, Hung TH, Li TK, Lin NC, Shen TL. Multiple domains of the *Tobacco mosaic virus* p126 protein can independently suppress local and systemic RNA silencing. *Mol Plant-Microbe Interact* **25**, 648-657 (2012).

2. Citovsky V, McLean BG, Zupan J, Zambryski PC. Phosphorylation of tobacco mosaic virus cell-to-cell movement protein by a developmentally-regulated plant cell wall-associated protein kinase. *Genes Dev* **7**, 904-910 (1993).

3. Saitou N, Nei M. The neighbor-joining method: a new method for reconstructing phylogenetic trees. *Mol Biol Evol* **4**, 406-425 (1987).

4. Felsenstein J. Confidence limits on phylogenies: an approach using the bootstrap. *Evolution* **39**, 783-791 (1985).

5. Zuckerkandl E, Pauling L. Evolutionary divergence and convergence in proteins. In: *Evolving Genes and Proteins* (eds Bryson V, Vogel HJ). Academic Press, New York (1965).

6. Tamura K, Stecher G, Peterson D, Filipski A, Kumar S. MEGA6: Molecular Evolutionary Genetics Analysis version 6.0. *Mol Biol Evol* **30**, 2725-2729 (2013).
