# Supplementary material for: Receptor-like kinase BAM1 facilitates early movement of the Tobacco mosaic virus
Source: Commun Biol. 2021 Apr 30;4:511. doi: 10.1038/s42003-021-02041-0 (PMC8087827; doi:10.1038/s42003-021-02041-0)
Supplement: Supplementary file 2 — Reporting Summary [file 42003_2021_2041_MOESM2_ESM.pdf]

nature research

Corresponding author(s): Phu-Tri Tran

Last updated by author(s): Mar 5, 2021

Reporting Summary

Nature Research wishes to improve the reproducibility of the work that we publish. This form provides structure for consistency and transparency in reporting. For further information on Nature Research policies, see our Editorial Policies and the Editorial Policy Checklist.

Statistics

For all statistical analyses, confirm that the following items are present in the figure legend, table legend, main text, or Methods section.

n/a	Confirmed	
☐	☒	The exact sample size (n) for each experimental group/condition, given as a discrete number and unit of measurement
☐	☒	A statement on whether measurements were taken from distinct samples or whether the same sample was measured repeatedly
☐	☒	The statistical test(s) used AND whether they are one- or two-sided<br>*Only common tests should be described solely by name; describe more complex techniques in the Methods section.*
☒	☐	A description of all covariates tested
☒	☐	A description of any assumptions or corrections, such as tests of normality and adjustment for multiple comparisons
☒	☐	A full description of the statistical parameters including central tendency (e.g. means) or other basic estimates (e.g. regression coefficient) AND variation (e.g. standard deviation) or associated estimates of uncertainty (e.g. confidence intervals)
☐	☒	For null hypothesis testing, the test statistic (e.g. F, t, r) with confidence intervals, effect sizes, degrees of freedom and P value noted<br>*Give P values as exact values whenever suitable.*
☒	☐	For Bayesian analysis, information on the choice of priors and Markov chain Monte Carlo settings
☒	☐	For hierarchical and complex designs, identification of the appropriate level for tests and full reporting of outcomes
☒	☐	Estimates of effect sizes (e.g. Cohen's d, Pearson's r), indicating how they were calculated

Our web collection on statistics for biologists contains articles on many of the points above.

Software and code

Policy information about availability of computer code

Data collection	Zen Blue 3.1 was used to collect images from confocal fluorescent microscope (LSM 900) as TIF format, ImageJ was then used for analyses of co-localization and movement distance. For qPCR, Ct value were exported from the MiniOpticon real-time PCR system (#CFB-3120, Bio-Rad) as excel format
Data analysis	one-tailed t-test, using Microsoft excel

For manuscripts utilizing custom algorithms or software that are central to the research but not yet described in published literature, software must be made available to editors and reviewers. We strongly encourage code deposition in a community repository (e.g. GitHub). See the Nature Research guidelines for submitting code & software for further information.

Data

Policy information about availability of data

All manuscripts must include a data availability statement. This statement should provide the following information, where applicable:

- Accession codes, unique identifiers, or web links for publicly available datasets
- A list of figures that have associated raw data
- A description of any restrictions on data availability

Figure 1c, figure 3, figure 4, figure 5a and b.

Field-specific reporting

Please select the one below that is the best fit for your research. If you are not sure, read the appropriate sections before making your selection.

☒ Life sciences ☐ Behavioural & social sciences ☐ Ecological, evolutionary & environmental sciences

For a reference copy of the document with all sections, see nature.com/documents/nr-reporting-summary-flat.pdf

Life sciences study design

All studies must disclose on these points even when the disclosure is negative.

Sample size	Samples size calculation is not available. The sample size were experimentally selected based on the variation of mean. For examples, in the qPCR experiments, n=6 for the arabidopsis Col-0, n=5 for the arabidopsis Ler-0 and n=3 for Nicotiana benthamiana. For other measurements: n=30 in co-localization, n=20 in movement of MP protein or TMV virus
Data exclusions	No exclusion
Replication	3 times. All replications are consistent
Randomization	the wildtype, mutant, and overexpression plants were grown in same culture condition. Tissue collection and measurement were conducted in same conditions.
Blinding	Blinding is not available. I am the only one conduct experiment

Reporting for specific materials, systems and methods

We require information from authors about some types of materials, experimental systems and methods used in many studies. Here, indicate whether each material, system or method listed is relevant to your study. If you are not sure if a list item applies to your research, read the appropriate section before selecting a response.

Materials & experimental systems

n/a	Involved in the study	
☐	☒	Antibodies
☒	☐	Eukaryotic cell lines
☒	☐	Palaeontology and archaeology
☒	☐	Animals and other organisms
☒	☐	Human research participants
☒	☐	Clinical data
☒	☐	Dual use research of concern

Methods

n/a	Involved in the study	
☒	☐	ChIP-seq
☒	☐	Flow cytometry
☒	☐	MRI-based neuroimaging

Antibodies

Antibodies used	Rabbit anti-Flag, Rabbit anti-Histidine, HRP goat anti rabbit
Validation	By western blotting using positive control - TMV MP with histidine tag and flag tag
